# Synthesis of artificial substrate based on inhibitor for detecting LSD1 activity

**DOI:** 10.3164/jcbn.20-9

**Published:** 2020-05-15

**Authors:** Yuhei Ohta, Mitsuyasu Kawaguchi, Naoya Ieda, Hidehiko Nakagawa

**Affiliations:** 1 Graduate School of Pharmaceutical Sciences, Nagoya City University, 3-1 Tanabedori, Mizuho-ku, Nagoya, Aichi 467-8603, Japan

**Keywords:** LSD1, epigenetics, enzymatic assay

## Abstract

Lysine methylation is one of the most important modification, which is regulated by histone lysine methyltransferases and histone lysine demethylases. Lysine-specific demethylase 1 (LSD1) specifically demethylates mono- and dimethyl-lysine on histone H3 (H3K4Me/Me_2_, H3K9Me/Me_2_) to control chromatin structure, resulting in transcriptional repression or activation of target genes. Furthermore, LSD1 is overexpressed in various cancers. Therefore, LSD1 inhibitors would be not only potential therapeutic agents for cancers but also chemical tools to research biological significance of LSD1 in physiological and pathological events. However, known assay methods to date have some inherent drawbacks. The development of simple method in detecting LSD1 activity has been indispensable to identify useful inhibitors. In this study, we designed and synthesized artificial substrates based on inhibitors of LSD1 to examine LSD1 activity by an absorption increment.

## Introduction

Histone modifications, including acetylation, methylation, phosphorylation and so on, change chromatin dynamics, and regulate gene and protein expressions, which is known as epigenetic regulation.^(1)^ Among the histone modifications, lysine (Lys) methylation is one of the most important modification, which is regulated by histone lysine methyltransferases (KMTs) and histone lysine demethylases (KDMs).^(2)^

**Fig. 1:**
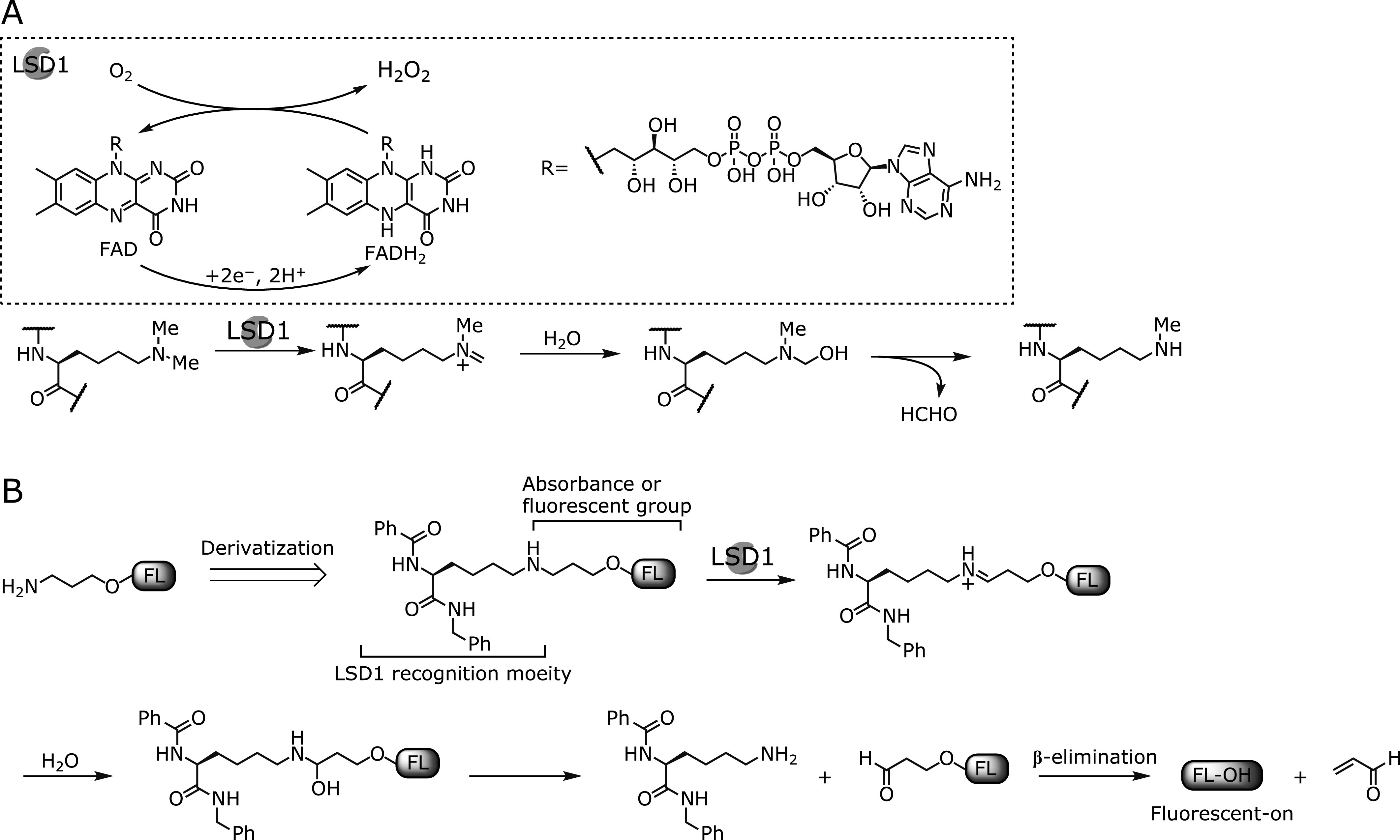
(A) FAD-dependent demethylation mechanism of methylated lysine by LSD1. (B) Our strategy to detect LSD1 activity through β-elimination.

Lysine-specific demethylase 1 (LSD1), also known as KDM1A, is firstly identified as a histone demethylase belonging to the amine oxidase family. LSD1 demethylates methylated Lys residues of target proteins through flavin-adenine dinucleotide (FAD)-dependent enzymatic activity, and generate demethylated product, hydrogen peroxide (H_2_O_2_) and formaldehyde (HCHO) (Fig. 1A).^(3)^ LSD1 specifically demethylates mono- and dimethyl-lysine on histone H3 (H3K4Me/Me_2_, H3K9Me/Me_2_) to control chromatin structure, resulting in transcriptional repression or activation of target genes.^(4–7)^ In addition, it is reported that LSD1 demethylates several non-histone proteins, such as p53, DNMT1, STAT3, and E2F1, whose functions and stabilities are altered by methylation levels.^(8–11)^ Furthermore, the expression level of LSD1 is related with some cancers. Indeed, it has been reported that LSD1 is overexpressed in various human cancers: including breast and colon cancers, and neuroblastoma.^(12–14)^ In such cancer cells, knockdown or pharmacological inhibition of LSD1 is reported to suppress tumor cell growth.^(15)^ Therefore, the inhibition of LSD1 activity would be not only a potential therapeutic strategy for cancers but also a chemical method to research biological significance of LSD1 in physiological and pathological events. So far, various LSD1 inhibitors have been developed and few of them has been used for clinical trials.^(16–20)^

To date, many kinds of assay method to detect LSD1 activity have been reported for screening LSD1 inhibitors. For example, mass spectrometry (MS)-based method is used to directly detect demethylated products to evaluate LSD1 activity.^(21,22)^ Also, indirect methods to evaluate LSD1 demethylase activity have been developed, in which H_2_O_2_ or HCHO, that are byproducts after LSD1 enzymatic reaction, are measured. In a coupled method with horseradish peroxidase (HRP) to detect generation of H_2_O_2_, products with fluorescence or chromophore are generated in H_2_O_2_ concentration-dependent manner.^(23,24)^ In contrast, in a coupled method with formaldehyde dehydrogenase (FDH) to detect HCHO, HCHO is metabolized by FDH with NAD^+^ as a co-enzyme, and generate NADH that are detectable through fluorescence and absorption changes.^(25)^ However, reported methods have some inherent drawbacks with regard to low-throughput and false positive or negative results due to a usage of other enzymes. To develop new LSD1 inhibitors, simple and reliable method for detecting LSD1 activity has been needed. Therefore, in this study, we have synthesized artificial LSD1 substrates based on a structure of LSD1 inhibitor and explored the reactivity of LSD1.

## Materials and Methods

### Chemicals

**Fig. 2:**
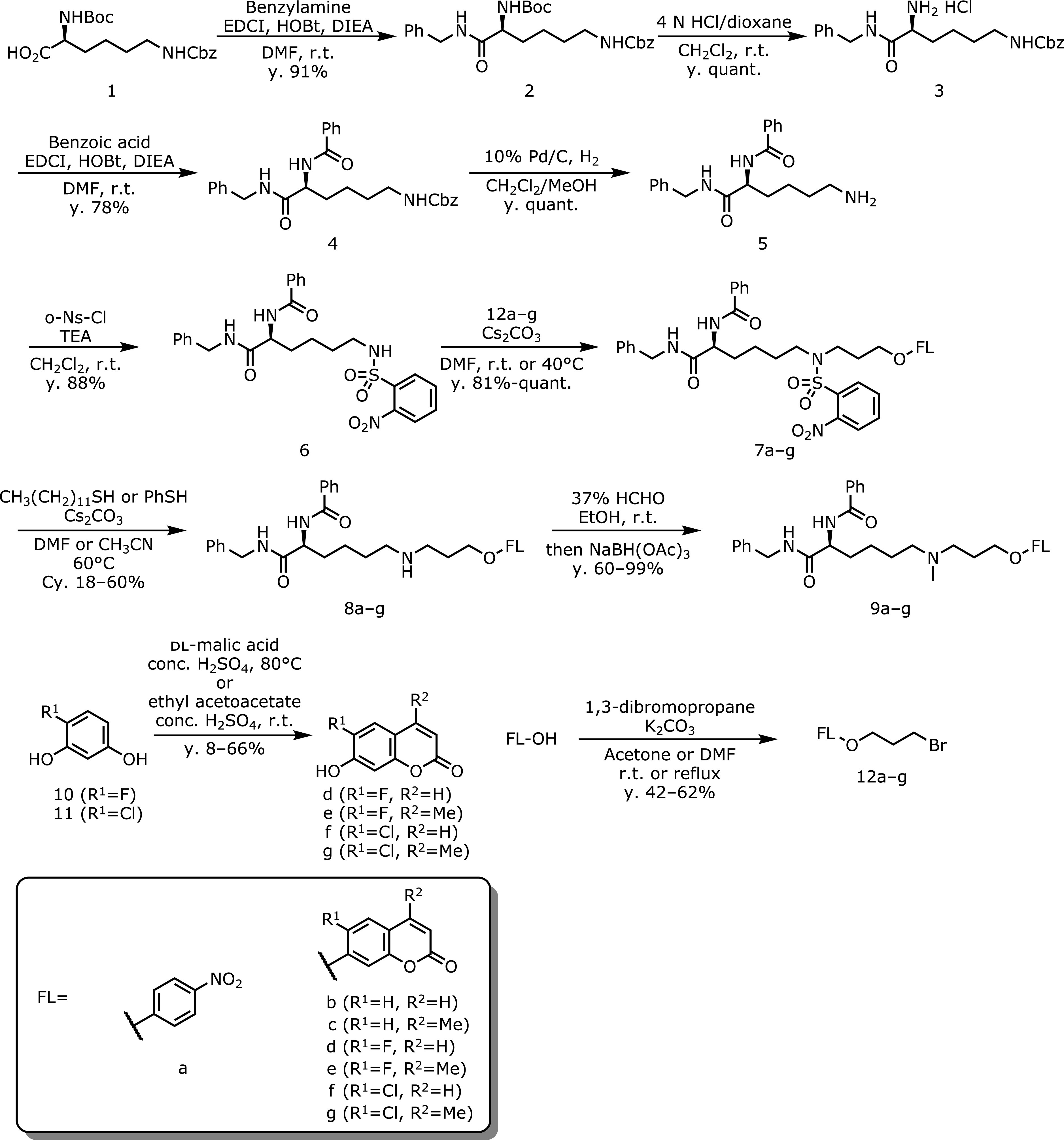
Synthesis of various LSD1 artificial substrates.

Proton nuclear magnetic resonance spectra (^1^H NMR) and carbon nuclear magnetic resonance spectra (^13^C NMR) were recorded on a Varian VNMRS 500 or JEOL JNM-ECZ500 spectrometer in the indicated solvent. Chemical shifts (δ) are reported in parts per million relative to the internal standard tetramethylsilane (TMS). Electrospray ionization (ESI) mass spectra were recorded on a JEOL JMS-T100LC mass spectrometer equipped with a nanospray ion source. Ultraviolet−visible-light absorption spectra were recorded on an Agilent 8453 spectrophotometer (Agilent Technologies Japan, Tokyo, Japan). Fluorescence spectra were recorded on an RF-5300PC fluorometer (Shimadzu, Kyoto, Japan). Analytical HPLC was performed with a Shimadzu pump system equipped with a reversed-phase ODS column (Inertsil ODS-3 4.6 mm × 150 mm, GL Science, Tokyo, Japan) at a flow rate of 1.0 ml/min. Microplate assay was performed on an ARVO X5 plate reader (PerkinElmer Japan, Kanagawa, Japan). LSD1 fluorometric drug discovery kit (BML-AK544-0001) containing recombinant LSD1 (BML-SE544-0050) and LSD1/HRP buffer (BML-KI566-0020) was purchased from Enzo Life Sciences, Inc. (Farmingdale, NY). All other reagents and solvents were purchased from Sigma-Aldrich (St. Louis, MO), Tokyo Chemical Industry Co., Ltd. (Tokyo, Japan), FUJIFILM Wako Pure Chemical Corporation (Osaka, Japan), Kanto Chemical Co., Inc. (Tokyo, Japan), Junsei Chemical Co., Ltd. (Tokyo, Japan), Nacalai Tesque (Kyoyo, Japan), or Watanabe Chemical Ind., Ltd. (Kyoto, Japan) and used without further purification. Flash column chromatography was performed using silica gel 60 (particle size 0.032–0.075) supplied by Taikoh-shoji (Aichi, Japan). LSD1 substrates were synthesized as shown in Fig. 2. Experimental procedures were shown in Supporting Information*. The purity of all synthesized compounds was assessed by HPLC and was ≥93% (recorded by 254 nm).

### Absorption and fluorescence spectroscopy

 Absorption and fluorescence spectra were measured in a quartz cuvette (10 × 4 × 45 mm) on Agilent 8543 and RF5300PC instruments, respectively. Spectra of LSD1 substrates were measured in Tris-HCl (pH 8.0), containing 150 mM NaCl. The final concentration of each LSD1 substrates was 5 µM (0.1% DMSO) except for 8a and 9a (10 µM, 0.1% DMSO). The fluorometer slit width was 1.5 nm for both excitation and emission, the sensitivity was set to high, and the excitation wavelength was 360 nm.

### Reactivity of LSD1 substrates for LSD1

LSD1 substrates (500 µM) and 10 ng/µl LSD1 were prepared by diluting each stock solution with LSD1/HRP buffer (Enzo Life Sciences, Inc.). LSD1 substrates solution (500 µM, 5 µl) and LSD1/HRP buffer (45 µl) were dispensed to each well (50 µl), then was added 10 ng/µl LSD1 (50 µl) or LSD1/HRP buffer (50 µl). The final volume of samples was all 100 µl [final conc; (LSD1 substrate) = 25 µM, (LSD1) = 5.0 ng/µl, 0.25% DMSO]. The assay plate was incubated for 3 h at 25°C. Absorption or fluorescence intensities were measured on plate reader at every 5 min (ARVO X5, λ_Abs_ = 405 nm or Ex = 355/40 nm, Em = 460/25 nm). Experiments were run in triplicate, and the results are shown as mean ± SD.

### Reactivity of 8a and 9a for LSD1 and denatured LSD1

Active LSD1 was denatured by heat at 90°C for 5 min. This assay was conducted using the same procedure as above.

### Inhibition assay for LSD1 activity using 8a and 9a, and HPLC analysis

500 µM 8a and 9a, LSD1 (10.3 ng/µl) and 8 µM GSK-LSD1 were prepared by diluting each stock solution with LSD1/HRP buffer. 10.3 ng/µl LSD1 (49 µl) and 8 µM GSK-LSD1 (1 µl) or LSD1/HRP buffer (1 µl) were dispensed to each well, then pre-incubated at 25°C for 30 min [conc. (LSD1) = 10 ng/µl, (GSK-LSD1) = 160 nM]. After pre-incubation, 500 µM 8a or 9a solution (5 µl) and LSD1/HRP buffer (45 µl) were added to each well. The final volume of samples was all 100 µl [final conc; (8a or 9a) = 25 µM, (LSD1) = 5.0 ng/µl]. The assay plate was incubated at 25°C for 3 h. Absorption were measured on plate reader at every 3 min (ARVO X5, λ_Abs_ = 405 nm). Experiments were run in triplicate, and the results are shown as mean ± SD.

After 3 h, each corresponding well containing 9a was analyzed by HPLC. HPLC conditions: A:B = 90:10 (0 min) to 0:100 (20 min) with a linear gradient, A = 0.1% TFA and B = 0.1% TFA CH_3_CN. Absorption was monitored at 320 nm.

### Detection of H_2_O_2_ for HRP coupled method

It was performed using a LSD1 fluorescent assay kit (Enzo Life Sciences, Inc., BML-AK544-0001). A mixture of CeLLestialTM Red (1/100), HRP (1/50), 9a (25 µM), and LSD1 (0.5 µg/well) were deposited in all wells. The assay plate was incubated at 25°C for 3 h. Absorption were measured on plate reader at every 3 min (ARVO X5, Ex. = 531/25 nm, Em. 595/60 nm). Experiments were run in triplicate.

### Statistical analysis

Data are presented as the mean ± SD (shown as error bars). Statistical significance was examined by means of Student’s *t* test or Bonferroni-type multiple *t* test by using GraphPad Prism6: *p<0.05, **p<0.01, ***p<0.001, ****p<0.0001, ns, not significant.

## Results and Discussions

### Our strategy for detecting LSD1 activity

 Fluorescence probes for monoamine oxidases (MAOs), which are FAD dependent amine oxidases same as LSD1, have been reported, whose detecting strategies are based on an amine oxidation followed by β-elimination mechanism.^(26–28)^ Generally, MAO florescence probes consist of amine, propyl linker, and fluorophore moieties, and are firstly oxidized by MAOs to generate imine, which is subsequently hydrolyzed by H_2_O to form aldehydes. Then, β-elimination reaction rapidly occurs and the fluorophore is released along with the generations of acrolein and ammonia, resulting in fluorescence increment. This strategy is so simple for detecting amine oxidases that such MAO fluorescence probes can be applicable to chemical screening. However, LSD1 probes based on this mechanism has not been developed.

Tranylcypromine (PCPA) is an irreversible and non-selective inhibitor for FAD-dependent amine oxidase.^(29)^ Focused on the mechanism-based inhibition of PCPA, LSD1 selective inhibitor has been developed.^(30–32)^ The lysine moiety of LSD1 specific inhibitors have been efficiently recognized by LSD1 because a methylated lysine residue is the substrate of LSD1, indicating that a Lys moiety functions as a career to deliver irreversible inhibitors to the LSD1 activity pocket.^(33)^

Based on the findings of MAO fluorescence probes and LSD1 selective inhibitors, we newly designed artificial LSD1 substrates. The substrates are composed of Lys derivatives conjugated to fluorophore or chromophore, via propyl linker. We anticipated that LSD1 would oxidize ɛ-amine group of modified Lys and convert it to an iminium intermediate, which is immediately hydrolyzed to form aldehyde, and the chromophore moiety and acrolein would be released through β-elimination reaction as the same mechanism of MAO fluorescence probes (Fig. 1B). We selected *p*-nitrophenol and coumarin derivatives as chromophore/fluorophore moieties, which have different molecular sizes, so that we can easily evaluate substrate reaction with LSD1 as an absorption and fluorescence increments, respectively.

### Synthesis of LSD1 substrates

LSD1 substrates bearing various chromophore moieties have been synthesized as shown in Fig. 2. Briefly, a common *N-*(2-nitrobenzenesulfonyl) (Nosyl) intermediate (6) was synthesized from Boc-Lys(Cbz)-OH (1) in five steps through condensation reactions and removals of protecting groups. The bromopropylated chromophores (12a–g) were synthesized through S_N_2 reactions of chromophores with 1,3-dibromopropane. Next, conjugation reaction of 6 and 12a–g gave 7a–g, which were deprotected by a treatment of thiol under basic condition to afford LSD1 pro-substrates 8a–g. Furthermore, *N*-methylated LSD1 substrates 9a–g were prepared by reductive amination of 8a–g. Structure of each LSD1 substrate was determined by ^1^H-NMR, ^13^C-NMR and HRMS.

### Photochemical properties of LSD1 substrates

Firstly, we measured absorption and fluorescence spectra of synthesized LSD1 substrates (Supplemental Fig. 1 and 2*). Absorption maxima (λ_max_) of LSD1 substrates were blue-shifted compared with original chromophores. Notably, *p*-nitrophenol-based LSD1 substrates (8a and 9a) showed no absorption at 405 nm, which is a λ_max_ of *p*-nitrophenol. In addition, as we expected, some fluorescence substrates (8b–g and 9b–g), showed no fluorescence upon excitation of coumarin (λ_max_ = 360 nm), in contrast, coumarin derivatives themselves showed strong fluorescence at the same excitation light. Therefore, these results suggest that when LSD1 substrates are demethylated/dealkylated by LSD1 and chromophores were released through β-elimination, we can detect absorption or fluorescence increments.

### Reactivity of synthesized LSD1 substrates with LSD1

**Fig. 3:**
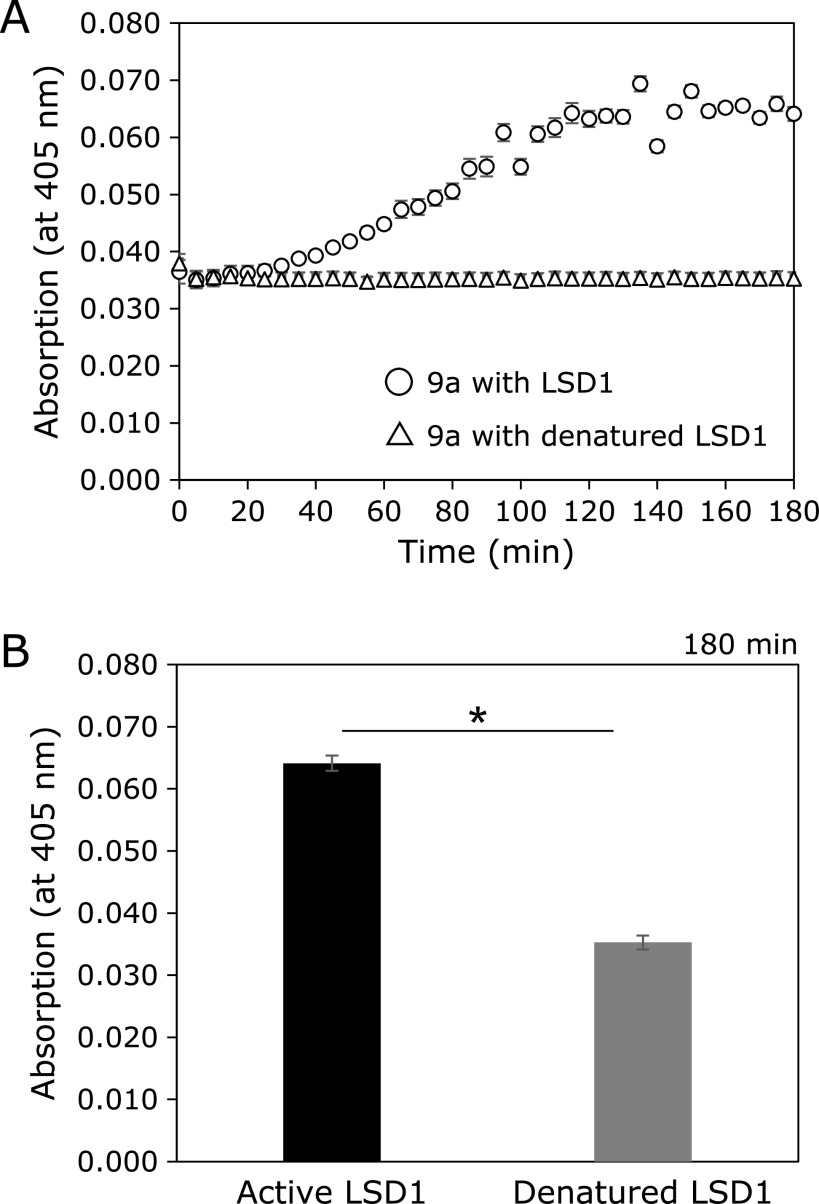
Enzymatic reaction of 9a with active LSD1 or heat-denatured LSD1 (90°C for 5 min). Enzymatic reactions were performed in LSD1/HRP buffer, containing 25 µM 9a, 5 ng/µl LSD1 (active or denatured). Absorption was measured with ARVO X5 (filters; 405/10 nm) every 5 min for 3 h at 25°C. The results are shown as mean ± SD. (*n* = 3). (A) Time-dependent curve (B) Absorption of 9a after enzymatic reaction for 180 min. *p<0.0001 (Student’s *t* test).

We next examined whether absorption or fluorescence increment can be observed concomitantly with LSD1 reaction (8a–g and 9a–g; LSD1, 5 ng/µl). However, LSD1 fluorescence substrates (8b–g and 9b–g) didn’t show fluorescence increment unfortunately (Supplemental Fig. 3*). On the other hand, LSD1 substrates with a *p*-nitrophenol moiety (8a and 9a) showed absorption increment at 405 nm, indicating that 8a and 9a were recognized and metabolized by LSD1 (Fig. 3 and Supplemental Fig. 4A*). As for the reason why LSD1 substrates containing coumarin derivatives did not exhibit fluorescence increment, we speculated that coumarin fluorophores were too large to accommodate the LSD1 catalytic pocket.

**Fig. 4:**
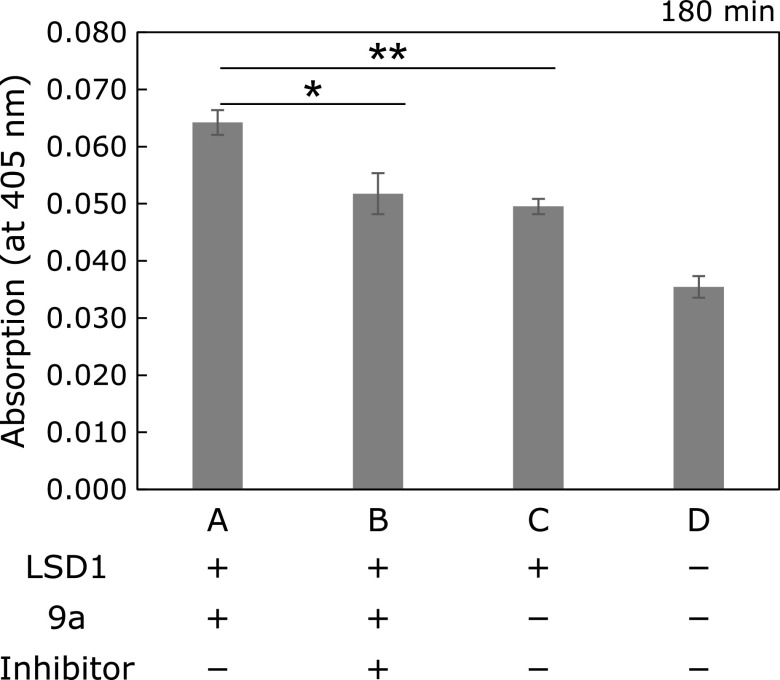
Enzymatic reaction of 9a with LSD1 in the presence or absence of LSD1 inhibitor. Enzymatic reactions were performed in LSD1/HRP buffer, containing 25 µM 9a, 5 ng/µl LSD1 after pre-incubation in the presence or absence of 160 nM GSK-LSD1 for 30 min. Absorption was measured with ARVO X5 (filters; 405/10 nm) after 3 h incubation at 25°C. The results are shown as mean ± SD (*n* = 3). *p<0.001, **p<0.0001 (Bonferroni-type multiple *t* test).

Furthermore, to confirm whether the absorption increment at 405 nm was taken place in an LSD1 catalytic activity-dependent manner, we conducted following two independent experiments. Firstly, we measured the time-dependent absorption increment using heat-denatured LSD1 and found that absorption increment was completely suppressed both in 8a and 9a assays (Fig. 3 and Supplemental Fig. 4A*). Secondly, when LSD1 was pre-incubated with the reported irreversible LSD1 inhibitor (GSK-LSD1) for 30 min,^(34)^ absorption increment of 9a was significantly suppressed compared with the control experiment [LSD1 (+), Inhibitor (–)] (Fig. 4), and, in contrast, that of 8a was not suppressed (Supplemental Fig. 4B*). We speculated that this difference of reactivity between 8a and 9a was probably due to the difference of their relative reactivity (*k*_cat_/*K*_m_) toward LSD1, because it has been reported that H3K4 peptide containing a dimethyl lysine residue is more likely to be a substrate for LSD1 than monomethyl one.^(35)^ These results indicate that LSD1 substrate 9a would be oxidized by LSD1 followed by *p*-nitrophenol release through β-elimination.

**Fig. 5:**
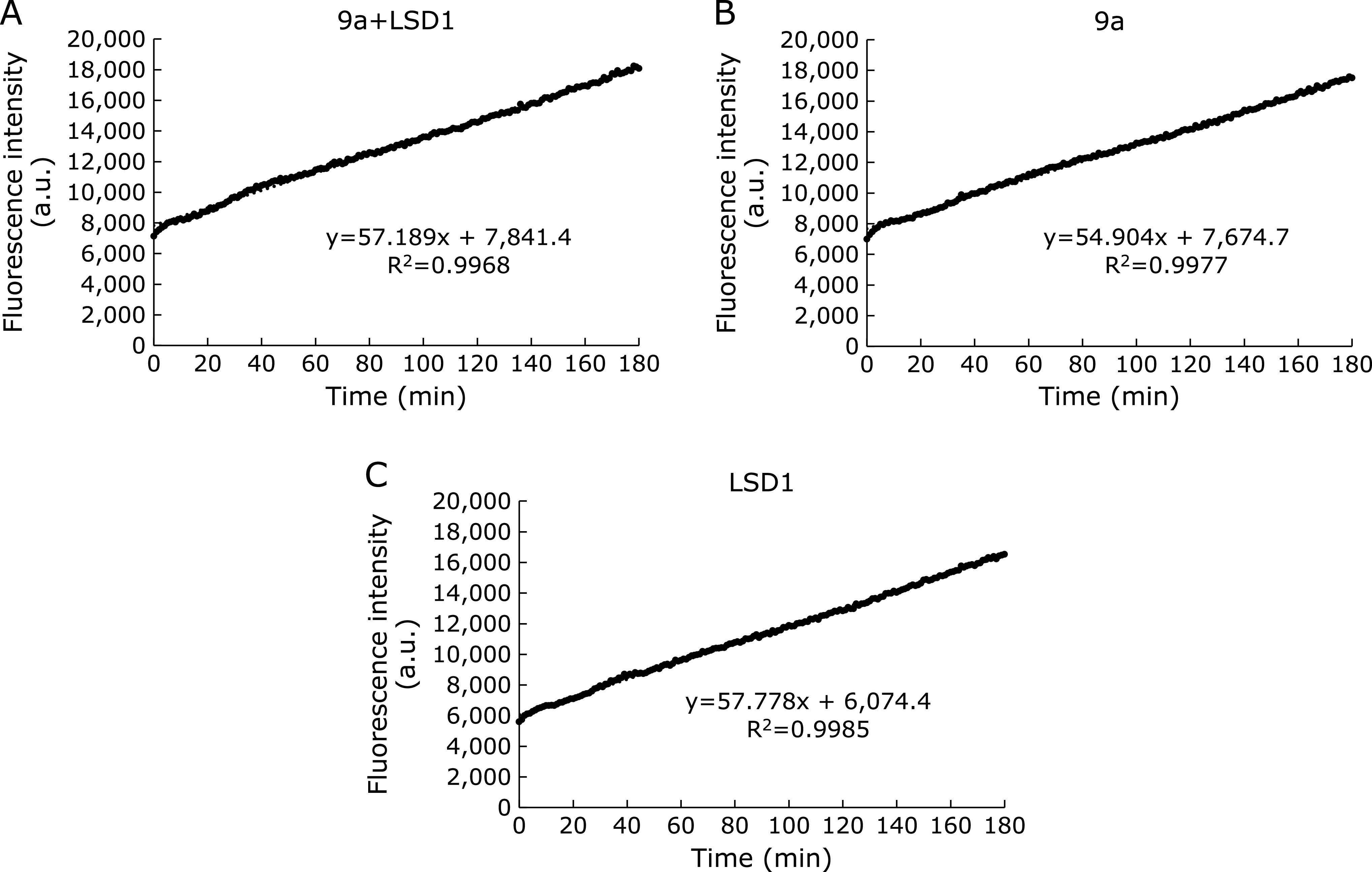
Detection of H_2_O_2_ in HRP assay. A mixture of CeLLestialTM Red, HRP, 9a (25 µM), and LSD1 (0.5 µg/well) were deposited in all wells. The assay plate was incubated at 25°C for 3 h. Absorption was measured with ARVO X5 (filters; Ex. = 531/25 nm, Em. 595/60 nm) every 3 min for 3 h. Experiments were run in triplicate.

In the result of a direct detection of *p*-nitrophenol in HPLC analysis, however, generation of *p*-nitrophenol in enzymatic reaction of 9a with LSD1 was not observed (Supplemental Fig. 5*). We considered that generated *p*-nitrophenol was lower than the detection limit in HPLC analysis due to very slow rate of metabolism of single amino acid derivatives in comparison with native-like long peptidyl substrate such as 20 mers.^(35)^ We also examined whether H_2_O_2_ generation can be observed during oxidation reaction of 9a with LSD1 (Fig. 1A). As a result, a generation of H_2_O_2_ was also not observed even in HRP-coupled fluorescence detection method (Fig. 5), whose detection limit is thought to be lower than an absorption detection with HPLC.

Although we did not get a direct evidence that 9a is oxidized by LSD1 and *p*-nitrophenol is released through β-elimination from above two experiments, suppression of absorption increment was reproductively observed in denatured LSD1 assay and pharmacological inhibition assay (Fig. 3 and 4). These biochemical experiments strongly supported that 9a became a substrate of LSD1 and should be oxidized by LSD1. In addition, because a compound having absorption at 405 nm is thought to be only *p*-nitrophenol in this assay condition, we think that small amount of generated *p*-nitrophenol and H_2_O_2_ are just less than detection limit.

In this study, we have found a novel LSD1 substrate 9a which can detect LSD1 activity as absorption increment. Since lacking of the evidence for a generation of *p*-nitrophenol and H_2_O_2_, it is possible that catalytic cycle cannot proceed many times, possibly partly due to the *in situ* formation of an FAD-acrolein adduct, in which acrolein is one of the enzymatic reaction products, or *p*-nitrophenol-LSD1 complex (product inhibition), which all may stop LSD1 enzymatic cycle (Fig. 1A). Because we could not elucidate the detailed mechanism of such potential suppression, further investigation is necessary to understand the catalytic mechanism of LSD1. However, substrate specificity of LSD1 obtained in this research would be helpful for developing new artificial substrates to directly detect LSD1 activity in one-step manner.

## Abbreviations

FAD: flavin-adenine dinucleotide
FDH: formaldehyde dehydrogenase
HRP: horseradish peroxidase
KDM: histone lysine demethylase
KMT: histone lysine methyltransferase
LSD1: lysine-specific demethylase 1
MAO: monoamine oxidase
