# Supplementary material for: Synthesis of artificial substrate based on inhibitor for detecting LSD1 activity
Source: J Clin Biochem Nutr. 2020 May 15;67(2):153–8. doi: 10.3164/jcbn.20-9 (PMC7533851; doi:10.3164/jcbn.20-9)
Supplement: Supporting Information [file jcbn20-9SI.pdf]

Supporting Information

Synthesis of artificial substrate based on inhibitor for detecting LSD1 activity

Yuhei Ohta, Mitsuyasu Kawaguchi, Naoya Ieda and Hidehiko Nakagawa*

Graduate School of Pharmaceutical Sciences, Nagoya City University, 3-1 Tanabedori, Mizuho-ku, Nagoya, Aichi 467-8603, Japan

E-mail: deco@phar.nagoya-cu.ac.jp

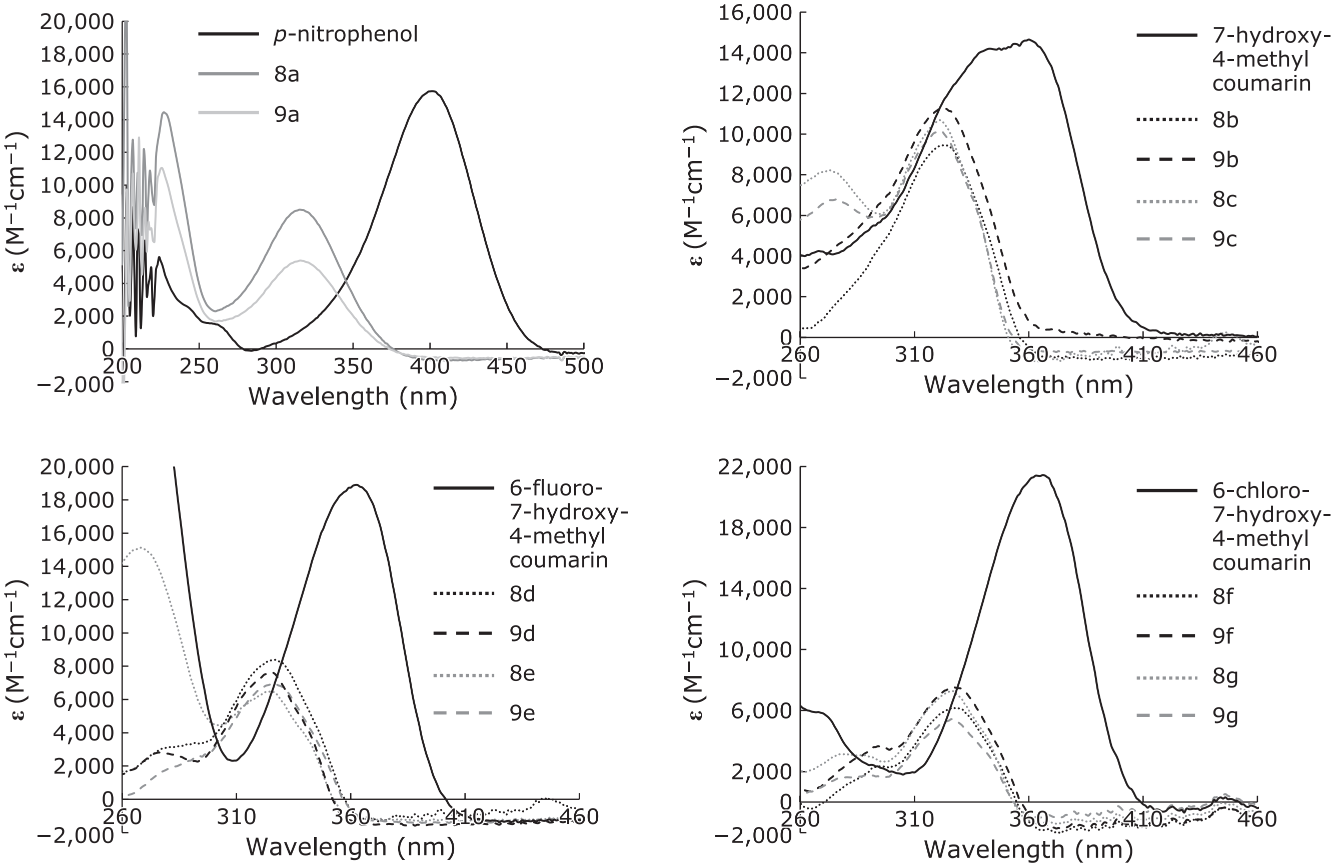

Supplemental Fig. 1. Absorption spectra of various 5 μM LSD1 substrates and reference compounds (*p*-nitrophenol, coumarin derivatives) expected for 8a, 9a, *p*-nitorophenol (10 μM) in 50 mM Tris-HCl buffer (pH 8.0).

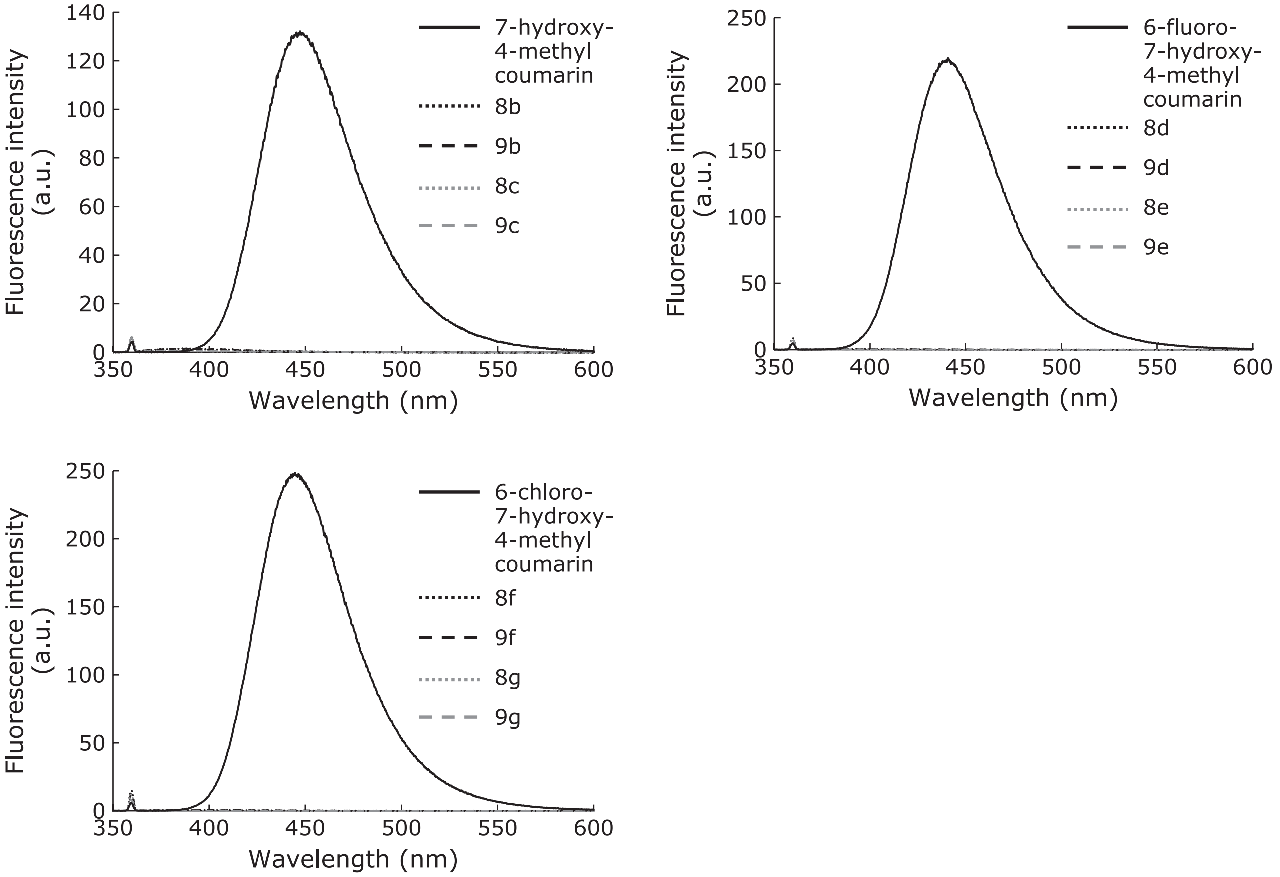

Supplemental Fig. 2. Fluorescence spectra of various 5 μM LSD1 substrates and reference compounds (coumarin derivatives) in 50 mM Tris-HCl buffer (pH 8.0). λ_{ex} = 360 nm.

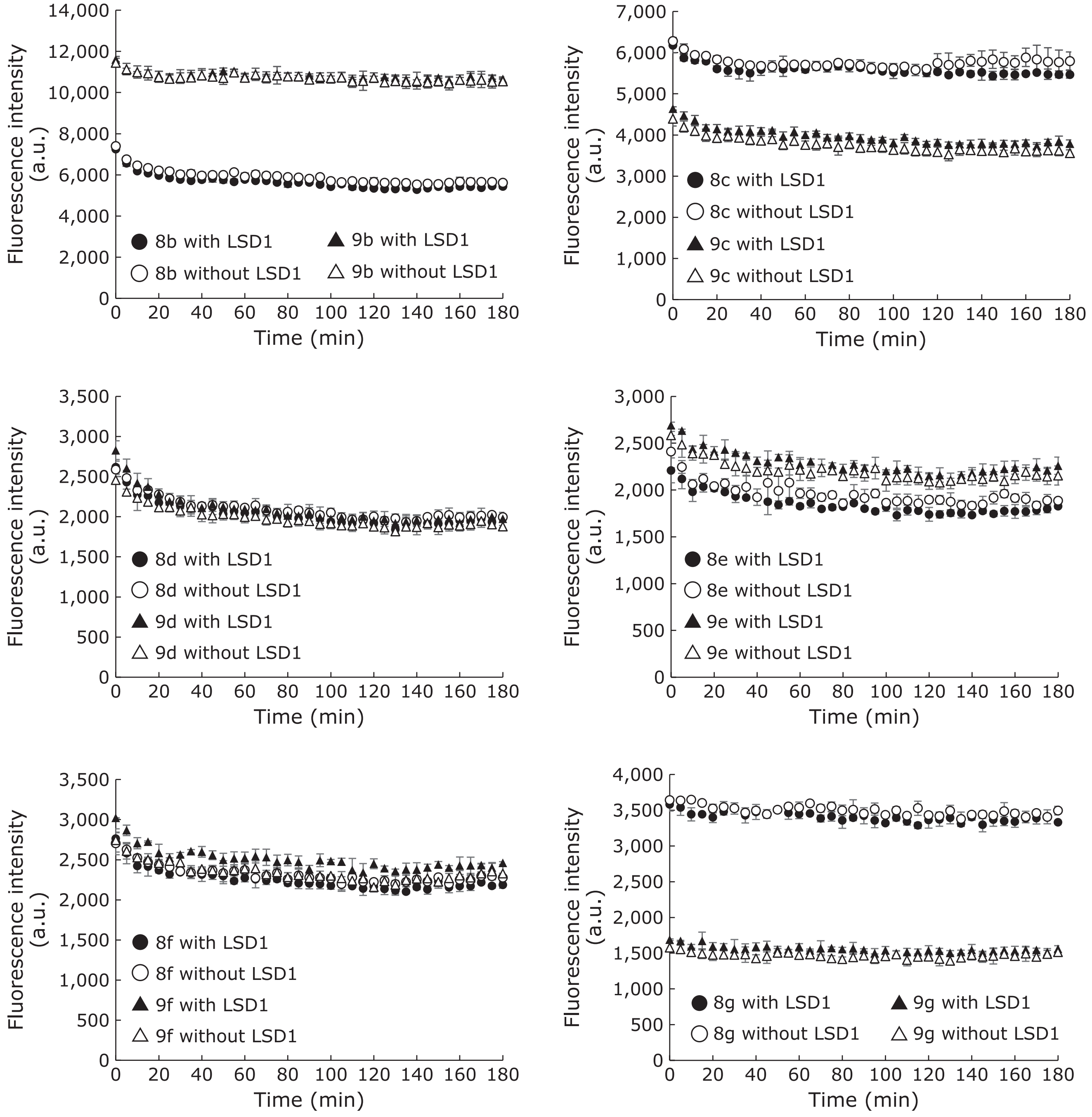

Supplemental Fig. 3. Enzymatic reactions of various LSD1 substrates with LSD1. Enzymatic reactions were performed in LSD1/HRP buffer, containing 25 μM LSD1 substrates in the presence or absence of 5 ng/μl LSD1. Fluorescence intensity was measured with ARVO X5 (filters; Ex. = 380/10 nm, Em. 460/20 nm) every 5 min for 3 h. The results are shown as mean ± SD (*n* = 3).

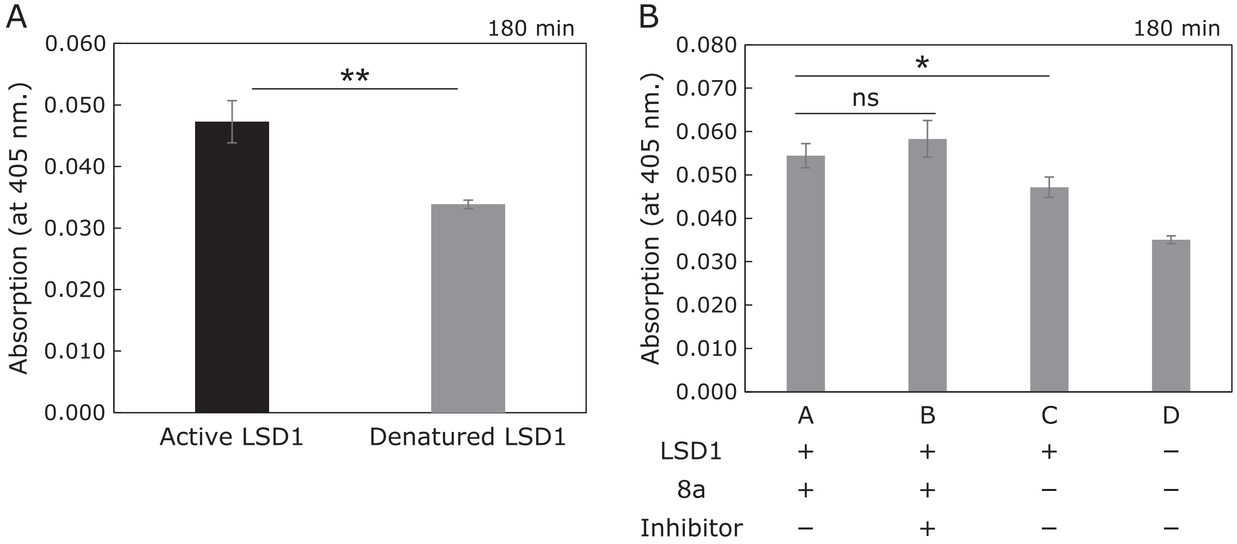

Supplemental Fig. 4. (A) Enzymatic reaction of 8a with active LSD1 or heat-denatured LSD1 (90°C for 5 min). Enzymatic reactions were performed in LSD1/HRP buffer, containing 25 μM 8a, 5 ng/μl LSD1 (active or denatured). Absorption was measured with ARVO X5 (filters; 405/10 nm) after 3 h incubation at 25°C. The results are shown as mean ± SD ($n = 3$). (B) Enzymatic reaction of 8a with LSD1 in the presence or absence of LSD1 inhibitor. Enzymatic reactions were performed in LSD1/HRP buffer, containing 25 μM 8a, 5 ng/μl LSD1 after pre-incubation in the presence or absence of 160 nM GSK-LSD1 for 30 min. Absorption was measured with ARVO X5 (filters; 405/10 nm) after 3 h incubation at 25°C. The results are shown as mean ± SD ($n = 3$). *$p<0.05$, **$p<0.01$, ns, not significant (Student's *t* test or Bonferroni-type multiple *t* test).

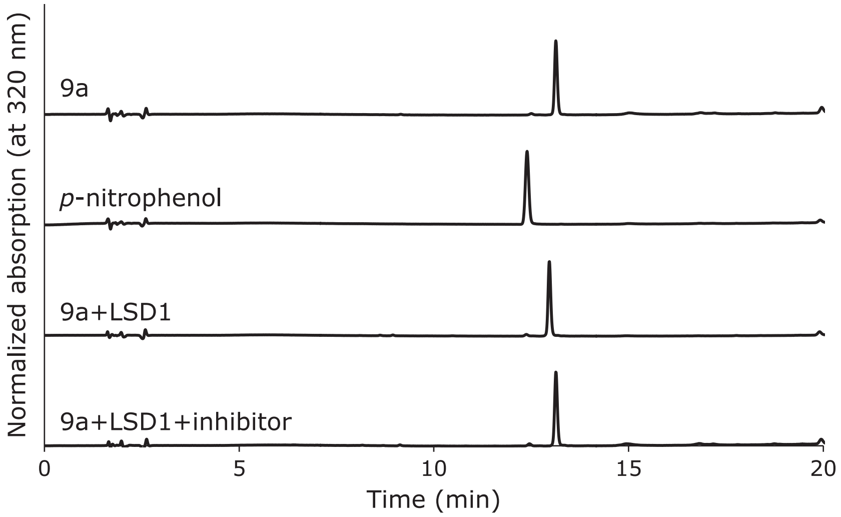

Supplemental Fig. 5. HPLC analysis of enzymatic reaction of LSD1 with 9a after 3 h. HPLC conditions: A:B = 90:10 (0 min) to 0:100 (20 min) with a linear gradient, A = 0.1% TFA and B = 0.1% TFA CH_3CN. Absorption at 320 nm was monitored.

Synthesis of *N*-Benzyl-6-(benzyloxycarbonyl)amino-2-[(tert-butoxycarbonyl)amino]hexanamide (2)

A solution of Boc-Lys(Cbz)-OH (1.00 g, 2.63 mmol), benzylamine (343 µl, 3.16 mmol, 1.2 eq.), EDCI · HCl (757 mg, 3.95 mmol, 1.5 eq.), HOBt · H_2O (605 mg, 3.95 mmol, 1.5 eq.) and DIEA (926 µl, 5.26 mmol, 2.0 eq.) in DMF (10 ml) was stirred at room temperature for 5 h. The reaction mixture was diluted with AcOEt, washed with 1 N HCl aq., sat. $NaHCO_3$ aq. and brine, and dried over Na_2SO_4. Filtration, evaporation *in vacuo*, purification by silica gel column chromatography (AcOEt/*n*-hexane = 1/2 to 1/1) to obtain 2 as white solid (1.12 g, yield; 91%); ^{1}H NMR (DMSO-d_6, 500 MHz, δ; ppm) 1.18–1.62 (m, 15H), 2.96 (d, 2H, *J* = 6.9 Hz), 3.87–3.91 (m, 1H), 4.27 (t, 2H, *J* = 5.7 Hz), 5.00 (s, 2H), 6.86 (d, 1H, *J* = 8.1 Hz), 7.20–7.38 (m, 11H), 8.29 (t, 1H, *J* = 6.3 Hz).

Synthesis of 2-Amino-*N*-benzyl-6-[(benzyloxycarbonyl)amino]hexanamide hydrochloride (3)

A solution of **2** (1.11 g, 2.36 mmol) and 4 N HCl/dioxane (5.90 ml, 23.6 mmol, 10 eq.) in CH_2Cl_2 (10 ml) was stirred at room temperature for 4 h, then evaporation *in vacuo* to obtain 3 as white solid (1.04 g, yield; quant.); ^{1}H NMR (DMSO-d_6, 500 MHz, δ; ppm) 1.25–1.32 (m, 4H), 1.37–1.42 (m, 2H), 1.72–1.77 (m, 2H), 2.96 (d, 2H, *J* = 6.5 Hz), 3.78 (br s, 1H), 4.34 (d, 2H, *J* = 5.8 Hz), 5.01 (s, 2H), 7.26–7.37 (m, 11H), 8.29 (br s, 3H), 9.07 (br s, 1H).

Synthesis of 2-(Benzenecarbonyl)amino-*N*-benzyl-6-[(benzyloxycarbonyl)amino]hexanamide (4)

A solution of 3 (1.03 g, 2.54 mmol), benzoic acid (372 mg, 3.05 mmol, 1.2 eq.), EDCI · HCl (730 mg, 3.81 mmol, 1.5 eq.), HOBt · H_2O (583 mg, 3.81 mmol, 1.5 eq.) and DIEA (1.33 ml, 7.62 mmol, 3.0 eq.) in DMF (10 ml) was stirred at room temperature for 6 h. The reaction mixture was diluted with AcOEt, washed with 1 N HCl aq., sat. $NaHCO_3$ aq. and brine, and dried over Na_2SO_4. Filtration, evaporation *in vacuo*, purification by silica gel column chromatography (AcOEt/*n*-hexane = 1/2 to 1/1) to obtain 4 as white solid (948 mg, yield; 79%); ^{1}H NMR (DMSO-d_6, 500 MHz, δ; ppm) 1.23–1.45 (m, 4H), 1.73–1.81 (m, 2H), 2.94–3.01 (m, 2H), 4.29 (d, 2H, *J* = 6.0 Hz), 4.42–4.46 (m, 1H), 4.98 (s, 2H), 7.20–7.55 (m, 14H), 7.91 (d, 2H, *J* = 7.3 Hz), 8.42–8.48 (m, 2H).

Synthesis of *N*-5-amino-1-({[(phenylmethyl) amino] carbonyl} pentyl) benzamide (5)

A solution of 4 (928 mg, 1.96 mmol) and 10% Pd/C (208 mg, 0.196 mmol, 0.10 eq.) in MeOH/CH_2Cl_2 (20/10 ml) was stirred at room temperature for 24 h under H_2. The reaction mixture was filtrated through Celite. Filtration *in vacuo* gave 5 as colorless amorphous (717 mg, yield; >100%); ^{1}H NMR (DMSO-d_6, 500 MHz, δ; ppm) 1.35–1.59 (m, 4H), 1.77–1.81 (m, 2H), 2.73–2.77 (m, 2H), 4.29 (d, 2H, *J* = 5.8 Hz), 4.45–4.49 (m, 1H), 7.21–7.33 (m, 5H), 7.43–7.56 (m, 3H), 7.78 (br s, 2H), 7.94 (d, 2H, *J* = 7.5 Hz), 8.45–8.55 (m, 2H).

Synthesis of *N*-{1-(benzylamino)-6-[(2-nitrophenyl)sulfonamide]-1-oxohexan-2-yl}benzamide (6)

To a solution of 5 (595 mg, 1.75 mmol) and Et_3N (488 µl, 3.50 mmol, 2.0 eq.) in CH_2Cl_2 (20 ml) was added slowly 2-nitrobenzenesulfonyl chloride (776 mg, 3.50 mmol, 2.0 eq.), then stirred at room temperature for 13 h. The reaction mixture was diluted with CH_2Cl_2, washed with water and brine, and dried over Na_2SO_4. Filtration, evaporation *in vacuo* and purification by silica gel flash column chromatography (AcOEt/*n*-hexane = 1/1 to 2/1) to obtain 6 as a pale gray solid (775 mg, yield; 88%); ^{1}H NMR (DMSO-*d6*, 500 MHz, δ; ppm) 1.23–1.48 (m, 4H), 1.66–1.75 (m, 2H), 2.85–2.89 (m, 2H), 4.28 (d, 2H, *J* = 5.9 Hz), 4.38–4.43 (m, 1H), 7.19–7.25 (m, 3H), 7.28–7.31 (m, 2H), 7.46 (t, 2H, *J* = 7.4 Hz), 7.53 (t, 1H, *J* = 7.4 Hz), 7.82–7.85 (m, 2H), 7.89–7.90 (m, 2H), 7.93–7.95 (m, 1H), 7.97–7.98 (m, 1H), 8.07 (t, 1H, *J* = 5.3 Hz), 8.40 (d, 1H, *J* = 7.8 Hz), 8.45 (t, 1H, *J* = 6.1 Hz).

General procedure for synthesis of 7a–7g

A solution of 6 (1.0 eq.), 12a–12g (1.5 eq.) and Cs_2CO_3 (1.5 eq.) in DMF was stirred at room temperature for 6–24 h. The reaction mixture was diluted with AcOEt, washed with water and brine, and dried over Na_2SO_4. Filtration, evaporation *in vacuo* and purification by silica gel flash column chromatography (AcOEt/*n*-hexane = 1/1 to 2/1) to obtain 7a–7g.

7a. colorless amorphous solid (156 mg, yield; 98%); ^{1}H NMR ($CDCl_3$, 500 MHz, δ; ppm) 1.42–1.45 (m, 2H), 1.60–1.73 (m, 3H), 1.80–1.86 (m, 1H), 1.95–1.99 (m, 1H), 2.01–2.09 (m, 2H), 3.32–3.37 (m, 2H), 3.48–3.52 (m, 2H), 4.01–4.04 (m, 2H), 4.47 (d, 2H, *J* = 4.0 Hz), 4.62 (d, 2H, *J* = 8.1 Hz), 6.56 (brs, 1H), 6.85–6.88 (m, 3H), 7.30–7.33 (m, 2H), 7.43–7.46 (m, 2H), 7.52–7.56 (m, 2H), 7.60–7.62 (m, 2H), 7.80 (d, 2H, *J* = 6.9 Hz), 7.97–7.99 (m, 1H), 8.16 (dd, 2H, *J* = 2.9 and 9.2 Hz).

7b. white solid (147 mg, yield; quant.); ^{1}H NMR ($CDCl_3$, 500 MHz, δ; ppm) 1.41–1.47 (m, 2H), 1.59–1.87 (m, 3H), 1.96–2.08 (m, 3H), 3.33–3.39 (m, 2H), 3.48–3.51 (m, 2H), 3.97 (d, 2H, *J* = 5.8 Hz), 4.46 (t, 2H, *J* = 5.8 Hz), 4.60–4.64 (m, 2H), 6.28 (d, 1H, *J* = 9.2 Hz), 6.59 (d, 1H, *J* = 5.8 Hz), 6.67 (d, 1H, *J* = 2.3 Hz), 6.77 (dd, 1H, *J* = 2.9 and 8.6 Hz), 6.88 (d, 1H, *J* = 8.0 Hz), 7.27–7.35 (m, 5H), 7.43–7.63 (m, 7H), 7.79–7.81 (m, 2H), 7.97–7.99 (m, 1H).

7c. colorless amorphous solid (200 mg, yield; 74%); ^{1}H NMR ($CDCl_3$, 500 MHz, δ; ppm) 1.40–1.47 (m, 2H), 1.61–1.87 (m, 3H), 1.96–2.09 (m, 3H), 2.39 (s, 3H), 3.33–3.37 (m, 2H), 3.50 (t, 2H, *J* = 6.5 Hz), 3.97 (t, 2H, *J* = 5.8 Hz), 4.46 (d, 2H, *J* = 5.9 Hz), 4.63 (d, 1H, *J* = 5.6 Hz), 6.13 (s, 2H), 6.65–6.67 (m, 2H), 6.79 (dd, 1H, *J* = 2.5 and 7.8 Hz), 6.90 (t, 2H,

J = 7.6 Hz), 7.27–7.32 (m, 10H), 7.42–7.61 (m, 7H), 7.80 (d, 2H, J = 7.2 Hz), 7.97–7.99 (m, 1H).

7d. white solid (209 mg, yield; quant.); ^{1}H NMR ($CDCl_3$, 500 MHz, δ; ppm) 1.41–1.47 (m, 2H), 1.62–1.88 (m, 3H), 1.96–2.02 (m, 1H), 2.06–2.11 (m, 2H), 3.31–3.41 (m, 2H), 3.51 (t, 2H, J = 6.8 Hz), 4.02 (t, 2H, J = 5.8 Hz), 4.42–4.50 (m, 2H), 4.62–4.66 (m, 1H), 6.31 (d, 1H, J = 9.7 Hz), 6.67 (t, 1H, J = 5.8 Hz), 6.75 (d, 1H, J = 7.0 Hz), 6.91 (d, 1H, J = 7.7 Hz), 7.11 (d, 1H, J = 10.3 Hz), 7.29–7.33 (m, 2H), 7.42–7.63 (m, 7H), 7.79–7.80 (m, 2H), 8.00–8.02 (m, 1H).

7e. white solid (97.1 mg, yield; 91%.); ^{1}H NMR ($CDCl_3$, 500 MHz, δ; ppm) 1.41–1.47 (m, 2H), 1.60–1.87 (m, 3H), 1.97–2.03 (m, 1H), 2.06–2.11 (m, 2H), 2.36 (d, 2H, J = 1.1 Hz), 3.31–3.40 (m, 2H), 3.50–3.53 (m, 2H), 4.03 (t, 2H, J = 5.8 Hz), 4.42–4.50 (m, 2H), 4.60–4.65 (m, 1H), 6.19 (d, 1H, J = 0.9 Hz), 6.62 (t, 1H, J = 5.8 Hz), 6.76 (d, 1H, J = 7.2 Hz), 6.89 (d, 1H, J = 7.6 Hz), 7.11 (d, 1H, J = 10.3 Hz), 7.30–7.33 (m, 2H), 7.42–7.64 (m, 6H), 7.80–7.81 (m, 2H), 8.00–8.03 (m, 1H).

7f. white solid (171 mg, yield; 81%.); ^{1}H NMR ($CDCl_3$, 500 MHz, δ; ppm) 1.41–1.49 (m, 2H), 1.60–1.87 (m, 3H), 1.96–2.02 (m, 1H), 2.09–2.13 (m, 2H), 3.32–3.42 (m, 2H), 3.54 (t, 2H, J = 6.7 Hz), 4.02 (t, 2H, J = 5.8 Hz), 4.46 (t, 2H, J = 6.0 Hz), 4.60–4.65 (m, 1H), 6.30 (d, 1H, J = 9.3 Hz), 6.60 (t, 1H, J = 5.8 Hz), 6.71 (s, 1H), 6.87 (d, 1H, J = 7.6 Hz), 7.30–7.33 (m, 2H), 7.42–7.47 (m, 3H), 7.50–7.61 (m, 4H), 7.77–7.82 (m, 2H), 8.00–8.03 (m, 1H).

7g. white solid (100 mg, yield; 87%.); ^{1}H NMR ($CDCl_3$, 500 MHz, δ; ppm) 1.42–1.47 (m, 2H), 1.62–1.88 (m, 3H), 1.97–2.02 (m, 1H), 2.08–2.13 (m, 2H), 2.38 (s, 3H), 3.32–3.42 (m, 2H), 3.54 (t, 2H, J = 7.5 Hz), 4.02 (t, 2H, J = 5.8 Hz), 4.43–4.50 (m, 2H), 4.61–4.65 (m, 1H), 6.17 (s, 1H), 6.63 (t, 1H, J = 5.9 Hz), 6.71 (s, 1H), 6.89 (d, 1H, J = 7.7 Hz), 7.30–7.33 (m, 2H), 7.42–7.45 (m, 2H), 7.50–7.62 (m, 5H), 7.79–7.82 (m, 2H), 8.00–8.04 (m, 1H).

General procedure for synthesis of 8a–8g

A solution of 7a–7g (1.0 eq.), thiophenol or 1-dodecanethiol (2.0–3.0 eq.) and Cs_2CO_3 (2.0–3.0 eq.) in CH_3CN was stirred at 60°C for 8–20 h. To the reaction mixture was diluted with CH_2Cl_2, washed with water and brine, and dried over Na_2SO_4. Filtration, evaporation *in vacuo* and purification by silica gel flash column chromatography (MeOH/ CH_2Cl_2 = 1/10 to 1/6) to obtain 8a–8g.

8a. pale yellow amorphous solid (50.7 mg, yield; 60%); ^{1}H NMR ($CDCl_3$, 500 MHz, δ; ppm) 1.45–1.60 (m, 4H), 1.77–1.83 (m, 1H), 1.93–2.03 (m, 3H), 2.59–2.67 (m, 2H), 2.75 (t, 2H, J = 6.9 Hz), 4.09 (t, 2H, J = 6.3 Hz), 4.42–4.52 (m, 2H), 4.65 (q, 1H, J = 7.5 Hz), 6.78 (t, 1H, J = 5.8 Hz), 6.86 (d, 1H, J = 8.0 Hz), 6.91–6.95 (m, 2H), 7.25–7.33 (m, 4H), 7.43 (t, 2H, J = 7.5 Hz), 7.51–7.54 (m, 1H), 7.77–7.79 (m, 2H), 8.17–8.19 (m, 2H); ^{13}C NMR ($CDCl_3$, 125 MHz, δ; ppm) 23.15, 29.43, 29.56, 29.70, 32.64, 43.66, 46.54, 48.31, 16.55, 53.34, 67.10, 114.41, 125.92, 127.02, 127.62, 127.70, 128.66, 128.76, 131.90, 133.73, 137.78, 141.44, 144.22, 164.00, 167.30, 171.41; HRMS (ESI$^+$): cacld: 519.26074, found: 519.25877 $[M + H]^+$ (–1.97 mDa); Purity by HPLC: 96.0% (254 nm).

8b. colorless amorphous solid (52.4 mg, yield; 55%); ^{1}H NMR ($CDCl_3$, 500 MHz, δ; ppm) 1.45–1.57 (m, 2H), 1.71–1.87 (m, 2H), 1.96–2.03 (m, 1H), 2.07–2.14 (m, 2H), 2.76–2.81 (m,2H), 2.93 (t, 2H, J = 7.0 Hz), 4.08 (t, 3H, J = 5.9 Hz), 4.39–4.48 (m, 2H), 4.71–4.76 (m, 1H), 6.23 (d, 1H, J = 9.5 Hz), 6.78–6.81 (m, 2H), 7.16 (d, 1H, J = 7.1 Hz), 7.27–7.32 (m, 5H), 7.40 (t, 2H, J = 7.5 Hz), 7.47–7.51 (m, 1H), 7.60 (d, 1H, J = 9.5 Hz), 7.76 (d, 2H, J = 7.2 Hz); ^{13}C NMR ($CDCl_3$, 125 MHz, δ; ppm) 22.84, 27.88, 32.95, 43.55, 46.21, 49.07, 53.16, 66.18, 101.45, 112.60, 112.79, 113.13, 127.08, 127.44, 127.72, 128.59, 128.64, 128.78, 131.91, 133.52, 137.97, 143.40, 155.79, 161.21, 161.90, 167.53, 171.53; HRMS (ESI$^+$): cacld: 542.26550, found: 542.26100 $[M + H]^+$ (–4.50 mDa); Purity by HPLC: 98.2% (254 nm).

8c. white solid (52.4 mg, yield; 55%); ^{1}H NMR (DMSO-*d6*, 500 MHz, δ; ppm) 1.31–1.53 (m, 4H), 1.73–1.83 (m, 2H), 1.89–1.96 (m, 2H), 2.39 (s, 3H), 2.64 (t, 2H, J = 7.3 Hz), 2.78 (t, 2H, J = 7.1 Hz), 4.13 (t, 2H, J = 6.2 Hz), 4.30 (d, 2H, J = 5.9 Hz), 4.45–4.49 (m, 1H), 6.21 (s, 1H), 6.94–6.97 (m, 2H), 7.21–7.31 (m, 5H), 7.47 (t, 2H, J = 7.7 Hz), 7.54 (t, 1H, J = 7.1 Hz), 7.68 (d, 1H, J = 8.5 Hz), 7.47 (d, 2H, J = 7.7 Hz), 8.47 (d, 1H, J = 8.0 Hz), 8.50 (t, 1H, J = 6.2 Hz); ^{13}C NMR (DMSO-*d6*, 125 MHz, δ; ppm) 18.21, 23.45, 27.70, 31.42, 42.08, 42.10, 45.28, 48.39, 53.60, 66.36, 101.24, 111.19, 112.54, 113.19, 126.57, 126.79, 127.11, 127.65, 128.25. 128.31, 131.140, 134.18, 139.53, 153.60, 154.80, 160.31, 161.69, 166.63, 172.06; HRMS (ESI$^+$): cacld: 556.28115, found: 556.28480 $[M + H]^+$ (3.66 mDa); Purity by HPLC: 96.0% (254 nm).

8d. colorless amorphous solid (61.3 mg, yield; 40%); ^{1}H NMR ($CDCl_3$ 500 MHz, δ; ppm) 1.47–1.63 (m, 2H), 1.89–1.96 (m, 1H), 1.99–2.06 (m, 3H), 2.40–2.45 (m, 2H), 2.95–3.04 (m, 2H), 3.22 (t, 2H, J = 6.9 Hz), 4.21 (t, 2H, J = 5.5 Hz), 4.33–4.44 (m, 2H), 4.85–4.89 (m, 1H), 6.29 (d, 1H, J = 9.2 Hz), 6.87 (d, 1H, J = 7.5 Hz), 7.10 (d, 1H, J = 10.3 Hz), 7.18–7.21 (m, 1H), 7.35 (t, 2H, J = 7.5 Hz), 7.46 (t, 1H, J = 7.5 Hz), 7.53 (d, 1H, J = 9.7 Hz), 7.59 (d, 1H, J = 7.5 Hz), 7.74 (d, 2H, J = 7.5 Hz), 8.23 (t, 1H, J = 6.2 Hz); ^{13}C NMR ($CDCl_3$, 125 MHz, δ; ppm) 14.13, 22.43, 25.60, 25.64, 33.00, 43.41, 15.44, 48.18, 52.97, 66.43, 102.46, 111.62, 111.68, 11323, 113.39, 114.61, 127.21, 127.27, 127.72, 128.56, 131.94, 133.32 138.19, 142.60,147.97, 149.84, 149.93, 151.29, 160.68, 167.82, 171.64; HRMS (ESI$^+$): cacld: 560.25607, found: 560.25488 $[M + H]^+$ (–1.19 mDa); Purity by HPLC: 95.2% (254 nm).

8e. colorless amorphous solid (51.2 mg, yield; 71%); ^{1}H NMR ($CDCl_3$, 500 MHz, δ; ppm) 1.43–1.71 (m, 4H), 1.87–2.05 (m, 4H), 2.33 (d, 3H, J = 1.1 Hz), 2.39–2.44 (m, 2H), 2.96–3.07 (m, 2H), 3.24 (t, 2H, J = 6.9 Hz), 4.21 (t, 2H, J = 5.5 Hz), 4.34–4.44 (m, 2H), 4.85–4.88 (m, 1H), 6.16 (s, 1H), 6.86 (d, 1H, J = 7.1 Hz), 6.97 (s, 1H), 7.18–7.25 (m, 2H), 7.34 (t, 2H, J = 7.7 Hz), 7.46 (t, 1H, J = 7.4 Hz), 7.59 (d, 1H, J = 8.0 Hz), 7.74 (d, 2H, J = 7.4 Hz), 8.22 (t, 1H, J = 5.5 Hz); ^{13}C NMR ($CDCl_3$, 125 MHz, δ; ppm) 18.70, 22.41, 25.55, 25.61, 32.95, 43.42, 45.53, 48.23, 52.97, 66.39, 102.47, 110.47, 110.64, 112.93, 112.98, 112.16, 127.20, 127.25, 127.71, 128.54, 131.93, 133.32, 138.20, 149.56, 149.66, 150.60, 151.87, 160.84, 167.84, 171.68;

HRMS (ESI^+): cacld: 574.27172, found: 574.26875 $[M + H]^+$ (–2.97 mDa); Purity by HPLC: 96.8% (254 nm).

8f. colorless amorphous solid (44.3 mg, yield; 34%); ^{1}H NMR ($CDCl_3$, 500 MHz, δ; ppm) 1.44–1.62 (m, 4H), 1.87–1.94 (m, 1H), 1.97–2.05 (m, 3H), 2.39–2.44 (m, 2H), 2.95–3.07 (m, 2H), 3.24 (t, 2H, J = 6.9 Hz), 4.19–4.22 (m, 2H), 4.34–4.46 (m, 2H), 4.83–4.88 (m, 1H), 6.28 (d, 1H, J = 9.8 Hz), 6.83 (s, 1H), 7.18–7.22 (m, 1H), 7.35 (t, 2H, J = 7.5 Hz), 7.41 (s, 1H), 7.45–7.48 (m, 1H), 7.52–7.56 (m, 2H), 7.74 (d, 2H, J = 7.5 Hz), 8.17–8.20 (m, 1H); ^{13}C NMR ($CDCl_3$, 125 MHz, δ; ppm) 14.13, 22.47, 22.68, 25.63, 25.68, 29.35, 29.64, 32.75, 43.40, 45.43, 48.19, 53.04, 66.42, 101.37, 112.83, 114.43, 119.18, 127.22, 127.25, 127.68, 128.15, 128.54, 131.89, 133.36, 138.16, 142.30, 154.05, 156.45, 160.42, 167.78, 171.65; HRMS (ESI^+): cacld: 576.22652, found: 576.22457 $[M + H]^+$ (–1.95 mDa); Purity by HPLC: 95.4% (254 nm).

8g. colorless amorphous solid (23.6 mg, yield; 32%); ^{1}H NMR ($CDCl_3$, 500 MHz, δ; ppm) 1.44–1.55 (m, 2H), 1.66–1.87 (m, 3H), 1.96–2.02 (m, 1H), 2.05–2.10 (m, 2H), 2.37 (s, 3H), 2.73 (d, 2H, J = 6.9 Hz), 2.89 (t, 2H, J = 6.9 Hz), 4.14 (t, 1H, J = 5.7 Hz), 4.41–4.48 (m, 2H), 4.69–4.73 (m, 1H), 6.15 (s, 1H), 6.87 (s, 1H), 7.07 (d, 1H, J = 6.9 Hz), 7.23–7.30 (m, 4H), 7.41 (d, 2H, J = 7.5 Hz), 7.54 (s, 1H), 7.78 (d, 2H, J = 7.5 Hz); ^{13}C NMR ($CDCl_3$, 125 MHz, δ; ppm) 18.64, 22.97, 28.25, 29.70, 32.86, 43.56, 46.42, 49.22, 53.28, 67.80, 101.21, 112.87, 113.81, 119.11, 125.30, 127.07, 127.44, 127.73, 128.57, 128.65, 131.82, 133.67, 137.98, 151.66, 153.55, 156.83, 160.70, 167.40, 171.54; HRMS (ESI^+): cacld: 590.24217, found: 590.24125 $[M + H]^+$ (–0.92 mDa); Purity by HPLC: 96.8% (254 nm).

General procedure for synthesis of 9a–9g

A solution of 8a–8g (1.0 eq.) and 37% HCHO aq. (4.0 eq.) in EtOH was stirred at room temperature for 1 h. To the resulting solution was added $NaBH(OAc)_3$ (2.0 eq.), then stirred at room temperature overnight. The reaction mixture was evaporated *in vacuo*, dissolved in CH_2Cl_2, washed with sat. $NaHCO_3$ aq. and brine, and dried over Na_2SO_4. Filtration, evaporation *in vacuo* and purification by silica gel flash column chromatography ($MeOH/CH_2Cl_2$ = 1/10 to 1/6) to obtain 9a–9g.

9a. pale yellow amorphous solid (28.0 mg, yield; 99%); ^{1}H NMR ($CDCl_3$, 500 MHz, δ; ppm) 1.43–1.49 (m, 2H), 1.59–1.66 (m, 2H), 1.76–1.83 (m, 1H), 1.96–2.04 (m, 3H), 2.32 (s, 3H), 2.46–2.56 (m, 2H), 2.62–2.68 (m, 2H), 4.08 (t, 2H, J = 5.8 Hz), 4.41–4.51 (m, 2H), 4.65 (d, 2H, J = 4.9 Hz), 6.91–6.95 (m, 4H), 7.27–7.33 (m, 6H), 7.42–7.45 (m, 2H), 7.50–7.53 (m, 1H), 7.78–7.80 (m, 2H), 8.16–8.19 (m, 2H); ^{13}C NMR ($CDCl_3$, 125 MHz, δ; ppm) 22.94, 26.19, 29.70, 32.50, 41.69, 43.64, 53.25, 53.94, 57.02, 66.69, 114.41, 125.94, 127.05, 127.57, 127.74, 128.65. 128.73, 131.89, 133.72, 137.87, 141.52, 163.86, 167.31, 171.40; HRMS (ESI^+): cacld: 533.27639, found: 533.28089 $[M + H]^+$ (4.50 mDa); Purity by HPLC: 98.3% (254 nm).

9b. colorless amorphous solid (20.6 mg, yield; 83%); 1H NMR ($CDCl_3$, 500 MHz, δ; ppm) 1.46–1.52 (m, 2H), 1.69–1.74 (m, 2H), 1.80–1.87 (m, 1H), 1.97–2.02 (m, 1H), 2.07–2.12 (m, 2H), 2.42 (s, 3H), 2.57–2.80 (m, 4H), 4.08 (t, 2H, J = 6.1 Hz), 4.42–4.51 (m, 2H), 4.66–4.70 (m, 1H), 6.24 (d, 1H, J = 9.5 Hz), 6.80–6.83 (m, 2H), 7.27–7.32 (m, 5H), 7.35 (d, 1H, J = 8.6 Hz), 7.43 (t, 2H, J = 7.5 Hz), 7.51 (t, 1H, J = 7.5 Hz), 7.62 (d, 1H, J = 9.5 Hz), 7.81 (d, 2H, J = 7.6 Hz); ^{13}C NMR ($CDCl_3$, 125 MHz, δ; ppm) 22.79, 25.60, 29.71, 32.42, 41.30, 43.59, 53.10, 53.94, 56.68, 66.22, 101.52, 112.69, 112.75, 113.22, 127.13, 127.47, 127.75, 128.60, 128.68, 128.85, 131.83, 133.76, 138.01, 143.40, 155.83, 161.22, 161.89, 167.39, 171.53; HRMS (ESI+): cacld: 556.28115, found: 556.27775 $[M + H]^+$ (–3.39 mDa); Purity by HPLC: 93.2% (254 nm).

9c. white solid (17.3 mg, yield; 99%); ^{1}H NMR (DMSO-*d6*, 500 MHz, δ; ppm) 1.28–1.49 (m, 4H), 1.72–1.91 (m, 4H), 2.28 (brs, 2H), 2.39 (s, 3H), 2.61 (brs, 2H), 4.10 (t, 2H, J = 6.2 Hz), 4.29 (d, 2H, J = 6.3 Hz), 4.44–4.48 (m, 1H), 6.21 (s, 1H), 6.94–6.96 (m, 2H), 7.20–7.32 (m, 5H), 7.46 (t, 2H, J = 7.5 Hz), 7.53 (t, 1H, J = 7.5 Hz), 7.65–7.67 (m, 1H), 7.90 (d, 2H, J = 8.0 Hz), 8.45 (d, 1H, J = 8.0 Hz), 8.49 (t, 1H, J = 6.2 Hz); ^{13}C NMR (DMSO-*d6*, 125 MHz, δ; ppm) 18.18, 23.51, 27.70, 31.46, 42.06, 53.16, 53.53, 66.39, 101.22, 111.16, 112.45, 113.16, 126.54, 126.75, 127.10, 127.60, 128.21. 128.27, 131.35, 134.16, 139.52, 153.53, 154.78, 160.27, 161.68, 166.55, 172.01; HRMS (ESI^+): cacld: 570.29680, found: 570.29839 $[M + H]^+$ (1.59 mDa); Purity by HPLC: 98.6% (254 nm).

9d. colorless amorphous solid (27.7 mg, yield; 86%); ^{1}H NMR ($CDCl_3$, 500 MHz, δ; ppm) 1.42–1.48 (m, 2H), 1.60–1.67 (m, 2H), 1.79–1.86 (m, 1H), 1.96–2.08 (m, 3H), 2.32 (s, 3H), 2.45–2.56 (m, 2H), 2.64–2.67 (m, 2H), 2.64 (t, 2H, J = 7.3 Hz), 2.78 (t, 2H, J = 7.1 Hz), 4.14 (t, 2H, J = 5.7 Hz), 4.40–4.51 (m, 2H), 4.67–4.71 (m, 1H), 6.29 (d, 1H, J = 9.8 Hz), 6.92 (d, 1H, J = 6.9 Hz), 7.07 (d, 1H, J = 9.5 Hz), 7.12–7.17 (m, 2H), 7.22–7.31 (m, 4H), 7.42 (t, 2H, J = 7.5 Hz), 7.50 (t, 1H, J = 7.5 Hz), 7.56 (d, 1H, J = 9.8 Hz), 7.79 (d, 2H, J = 7.5 Hz); ^{13}C NMR ($CDCl_3$, 125 MHz, δ; ppm) 22.70, 23.05, 25.92, 26.23, 29.36, 29.71, 32.59, 41.60, 43.58, 53.30, 53.67, 57.00, 67.46, 102.36, 111.33, 111.39, 113.19, 113.35, 114.38, 127.11, 127.46, 127.72, 128.59, 128.66, 131.79, 138.01, 142.72, 148.16, 150.12, 150.43, 150.54, 151.54, 160.87, 167.36, 171.60; HRMS (ESI^+): cacld: 574.27172, found: 574.27126 $[M + H]^+$ (–0.47 mDa); Purity by HPLC: 96.4% (254 nm).

9e. colorless amorphous solid (21.6 mg, yield; 80%);^{1}H NMR ($CDCl_3$, 500 MHz, δ; ppm) 1.43–1.49 (m, 2H), 1.63–1.68 (m, 2H), 1.80–1.87 (m, 1H), 1.97–2.02 (m, 1H), 2.02–2.10 (m, 2H), 2.35 (s, 6H), 2.48–2.59 (m, 2H), 2.67–2.75 (m, 2H), 4.14 (t, 2H, J = 6.3 Hz), 4.39–4.51 (m, 2H), 4.69–4.73 (m, 1H), 6.17 (s, 1H), 6.91 (d, 1H, J = 7.0 Hz), 7.12 (d, 1H, J = 8.0 Hz), 7.23–7.29 (m, 5H), 7.41 (t, 2H, J = 7.5 Hz), 7.49 (t, 1H, J = 7.0 Hz), 7.79 (d, 2H, J = 7.4 Hz); ^{13}C NMR ($CDCl_3$, 125 MHz, δ; ppm) 18.72, 23.00, 25.74, 25.98, 32.56, 41.46, 43.55, 53.25, 53.63, 56.86, 67.36, 102.40, 110.45, 110.61, 112.68, 112.74, 113.01, 127.12, 127.43, 127.71, 128.57, 128.65, 131.78, 133.79, 138.06, 148.10, 150.05, 150.10, 150.72, 151.97, 160.99, 167.37, 171.64; HRMS (ESI^+): cacld: 588.28737, found: 588.28704 $[M + H]^+$ (–0.33 mDa); Purity by HPLC: 98.9% (254 nm).

9f. colorless amorphous solid (16.5 mg, yield; 78%); ^{1}H NMR ($CDCl_3$, 500 MHz, δ; ppm) 1.46–1.52 (m, 2H), 1.77–2.03 (m, 4H), 2.20–2.23 (m, 2H), 2.56 (s, 3H), 2.73–2.80 (m, 2H), 2.96 (t, 2H, J = 7.4 Hz), 4.16 (t, 2H, J = 5.7 Hz), 4.39–4.50 (m,

2H), 4.72–4.76 (m, 1H), 6.29 (d, 1H, $J = 9.8$ Hz), 6.86 (s, 1H), 7.23–7.31 (m, 4H), 7.41 (t, 2H, $J = 7.5$ Hz), 7.45 (s, 1H), 7.49 (t, 2H, $J = 7.5$ Hz), 7.56 (d, 1H, $J = 9.8$ Hz), 7.81 (d, 2H, $J = 7.5$ Hz); ^{13}C NMR ($CDCl_3$, 125 MHz, δ; ppm) 22.67, 24.88, 29.69, 32.34, 40.95, 43.50, 53.03, 53.54, 56.45, 66.91, 101.29, 112.82, 114.44, 119.27, 127.16, 127.36, 127.73, 128.22, 128.56, 128.61, 131.78, 133.71, 138.12, 142.31, 154.14, 156.66, 160.47, 167.42, 171.58; HRMS (ESI$^+$): cacld: 590.24217, found: 590.24064 $[M + H]^+$ (–1.53 mDa); Purity by HPLC: 95.7% (254 nm).

9g. colorless amorphous solid (8.1 mg, yield; 60%); ^{1}H NMR ($CDCl_3$, 500 MHz, δ; ppm) 1.47–1.58 (m, 2H), 1.79–1.90 (m, 4H), 2.17–2.27 (m, 2H), 2.37 (s, 3H), 2.59 (s, 3H), 2.77–2.84 (m, 2H), 2.98–3.03 (m, 2H), 4.16 (t, 2H, $J = 5.7$ Hz), 4.40–4.51 (m, 2H), 4.70–4.75 (m, 1H), 6.17 (s, 1H), 6.86 (s, 1H), 7.21–7.30 (m, 4H), 7.40–7.43 (m, 3H), 7.48–7.51 (m, 1H), 7.56 (s, 1H), 7.82 (d, 2H, $J = 7.5$ Hz); ^{13}C NMR ($CDCl_3$, 125 MHz, δ; ppm) 18.34, 22.58, 24.79, 29.68, 32.34, 40.97, 13.54, 52.95, 53.62, 56.46, 66.78, 101.32, 113.09, 114.11, 119.06, 125.43, 127.15, 127.37, 127.75, 128.57, 128.62, 131.79, 133.71, 138.11, 151.58, 153.53, 156.42, 160.58, 167.43, 171.54; HRMS (ESI$^+$): cacld: 604.25782, found: 604.25815 $[M + H]^+$ (0.33 mDa); Purity by HPLC: 96.7% (254 nm).

General procedure for synthesis of d, f

A solution of resorcinol derivatives (1.0 eq.) and DL-malic acid (1.1 eq) in conc. H_2SO_4 was stirred at 80°C overnight. Afterwards, to the resulting mixture was added iced water. The formed precipitate was filtered and washed water and purified by recrystallization with acetone and/or silica gel column chromatography (AcOEt/ *n*-hexane = 1/2 to 1/1) to obtain d, f.

d. white powder (130 mg, yield; 9.3%); ^{1}H NMR ($CDCl_3$, 500 MHz, δ; ppm) 5.59 (d, 2H, $J = 4.6$ Hz), 6.33 (d, 1H, $J = 9.2$ Hz), 6.99 (d, 1H, $J = 7.5$ Hz), 7.20 (d, 1H, $J = 9.7$ Hz), 7.58 (d, 1H, $J = 9.8$ Hz).

f. gray solid (149 mg, yield; 7.6%); ^{1}H NMR ($CDCl_3$, 500 MHz, δ; ppm) 6.31 (t, 1H, $J = 9.8$ Hz), 7.00 (s, 1H), 7.47 (s, 1H), 7.58 (d, 1H, $J = 9.8$ Hz).

General procedure for synthesis of e, g

Conc. H_2SO_4 was added slowly to a mixture of resorcinol derivatives (1.0 eq.) and ethyl acetoacetate (1.1 eq.) at 0°C. The mixture was stirred at room temperature overnight. Afterwards, to the resulting mixture was added iced water. The formed precipitate was filtered and washed water to obtain e, g.

e. gray powder (73.7 mg, yield; 24%); ^{1}H NMR ($CDCl_3$, 500 MHz, δ; ppm) 2.37 (d, 3H, $J = 2.2$ Hz), 6.19 (s, 1H), 6.99 (d, 1H, $J = 7.5$ Hz), 7.64 (d, 1H, $J = 10.5$ Hz).

g. off-white solid (1.40 g, yield; 66%); ^{1}H NMR ($CDCl_3$, 500 MHz, δ; ppm) 2.39 (s, 3H), 6.19 (s, 1H), 7.00 (s, 1H), 7.56 (s, 1H).

General procedure for synthesis of 12a–12g

A solution was a–g, 1,3-dibromopropane (1.5 eq.) and K_2CO_3 (1.5 eq.) in DMF or acetone was stirred at room temperature or reflux temperature for 12–24 h. The reaction mixture was diluted with AcOEt/water, washed with brine, dried over Na_2SO_4, filtered and evaporated *in vacuo*. The residue was purified by silica gel column chromatography (AcOEt/*n*-hexane = 1/4 to 1/2) to obtain 12a–12g.

12a. white solid (321 mg, yield; 42%); ^{1}H NMR ($CDCl_3$, 500 MHz, δ; ppm) 2.35–2.41 (m, 2H), 3.60–3.63 (m, 2H), 4.21–4.23 (m, 2H), 6.97–6.99 (m, 2H), 8.21–8.23 (m, 2H).

12b. white solid (107 mg, yield; 61%); ^{1}H NMR ($CDCl_3$, 500 MHz, δ; ppm) 2.34–2.39 (m, 2H), 3.62 (t, 2H, $J = 6.3$ Hz), 4.18 (t, 2H, $J = 5.7$ Hz), 6.27 (dd, 1H, $J = 1.0$ and 9.5 Hz), 6.84–6.86 (m, 2H), 7.38 (d, 1H, $J = 8.0$ Hz), 7.64 (d, 1H, $J = 9.5$ Hz).

12c. white solid (yield; 62%); ^{1}H NMR ($CDCl_3$, 500 MHz, δ; ppm) 2.34–2.38 (m, 2H), 3.40 (s, 3H), 3.62 (t, 2H, $J = 6.9$ Hz), 4.18 (t, 2H, $J = 6.3$ Hz), 6.14 (s, 1H), 6.83 (d, 1H, $J = 2.5$ Hz), 6.87 (dd, 1H, $J = 2.5$ and 8.8 Hz), 7.50 (d, 1H, $J = 8.8$ Hz).

12d. white solid (123 mg, yield; 61%); ^{1}H NMR ($CDCl_3$, 500 MHz, δ; ppm) 2.38–2.43 (m, 2H), 3.64 (t, 2H, $J = 6.3$ Hz), 4.24 (t, 2H, $J = 5.7$ Hz), 6.33 (d, 1H, $J = 9.6$ Hz), 6.93 (d, 1H, $J = 8.7$ Hz), 7.18 (d, 1H, $J = 8.7$ Hz), 7.59 (d, 1H, $J = 9.6$ Hz).

12e. white solid (68.1 mg, yield; 58%); ^{1}H NMR ($CDCl_3$, 500 MHz, δ; ppm) 2.38 (s, 3H), 2.39–2.43 (m, 2H), 3.62–3.65 (m, 2H), 4.23–4.26 (m, 2H), 6.21 (s, 1H), 6.93 (dd, 1H, $J = 2.0$ and 7.1 Hz), 7.27–7.30 (m, 1H).

12f. white solid (139 mg, ield; 58%); ^{1}H NMR ($CDCl_3$, 500 MHz, δ; ppm) 2.40–2.45 (m, 2H), 3.67 (t, 2H, $J = 6.3$ Hz), 4.24 (t, 2H, $J = 5.7$ Hz), 6.31 (d, 2H, $J = 9.6$ Hz), 6.89 (s, 1H), 7.49 (s, 1H), 7.59 (d, 1H, $J = 9.6$ Hz).

12g. white solid (71.2 mg, yield; 47%); ^{1}H NMR ($CDCl_3$, 500 MHz, δ; ppm) 2.39 (s, 1H), 2.41–2.44 (m, 2H), 3.70 (t, 2H, $J = 6.3$ Hz), 4.23 (t, 2H, $J = 5.7$ Hz), 6.18 (s, 1H), 6.88 (s, 1H), 7.58 (s, 1H).
