# Supplementary material for: Synthesis of artificial substrate based on inhibitor for detecting LSD1 activity
Source: J Clin Biochem Nutr. 2020 May 15;67(2):153–8. doi: 10.3164/jcbn.20-9 (PMC7533851; doi:10.3164/jcbn.20-9)
Supplement: Supplemental Figure 1 [file jcbn20-9sf01.pdf]

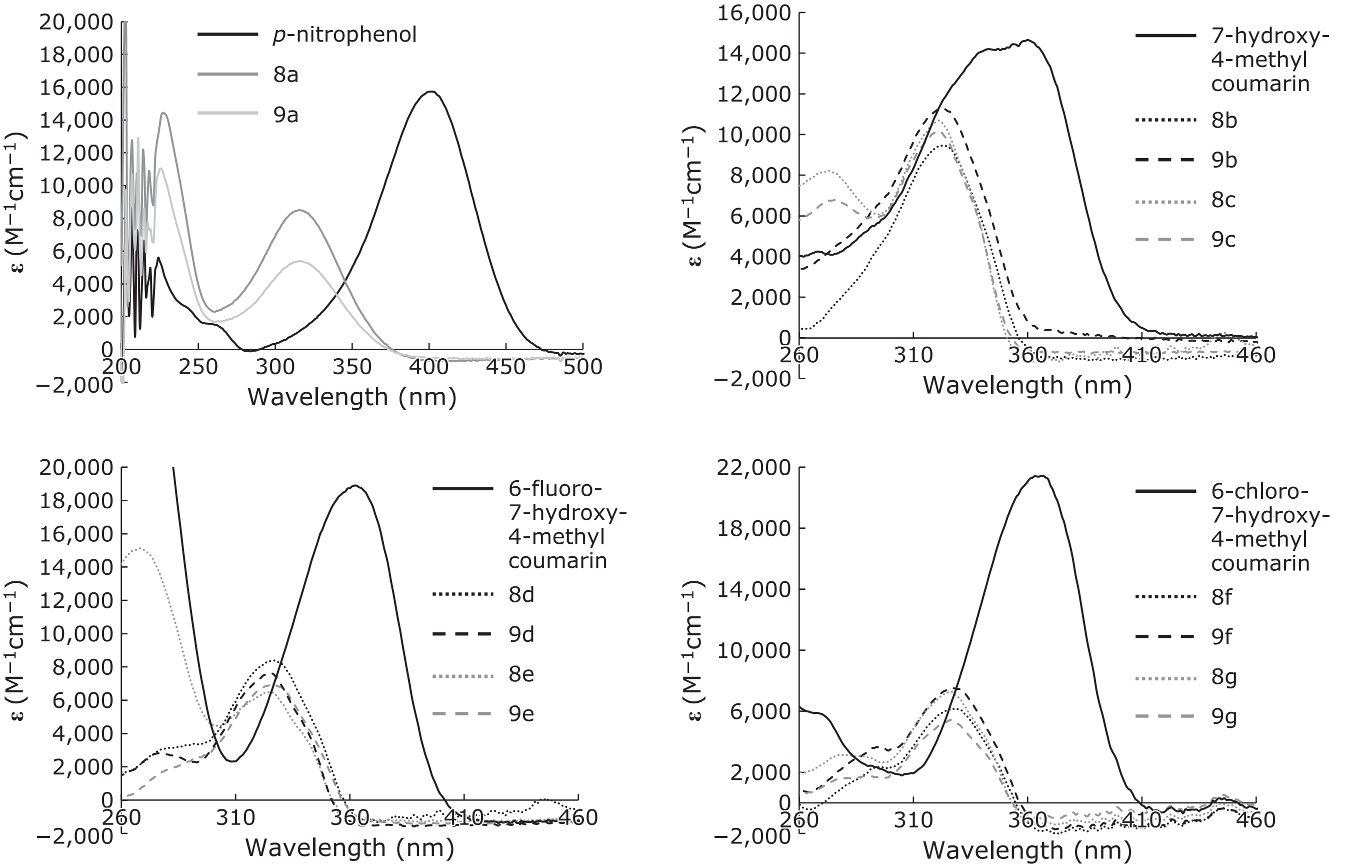

Supplemental Fig. 1. Absorption spectra of various 5 μM LSD1 substrates and reference compounds (*p*-nitrophenol, coumarin derivatives) expected for 8a, 9a, *p*-nitorophenol (10 μM) in 50 mM Tris-HCl buffer (pH 8.0).
