# Supplementary material for: Synthesis of artificial substrate based on inhibitor for detecting LSD1 activity
Source: J Clin Biochem Nutr. 2020 May 15;67(2):153–8. doi: 10.3164/jcbn.20-9 (PMC7533851; doi:10.3164/jcbn.20-9)
Supplement: Supplemental Figure 2 [file jcbn20-9sf02.pdf]

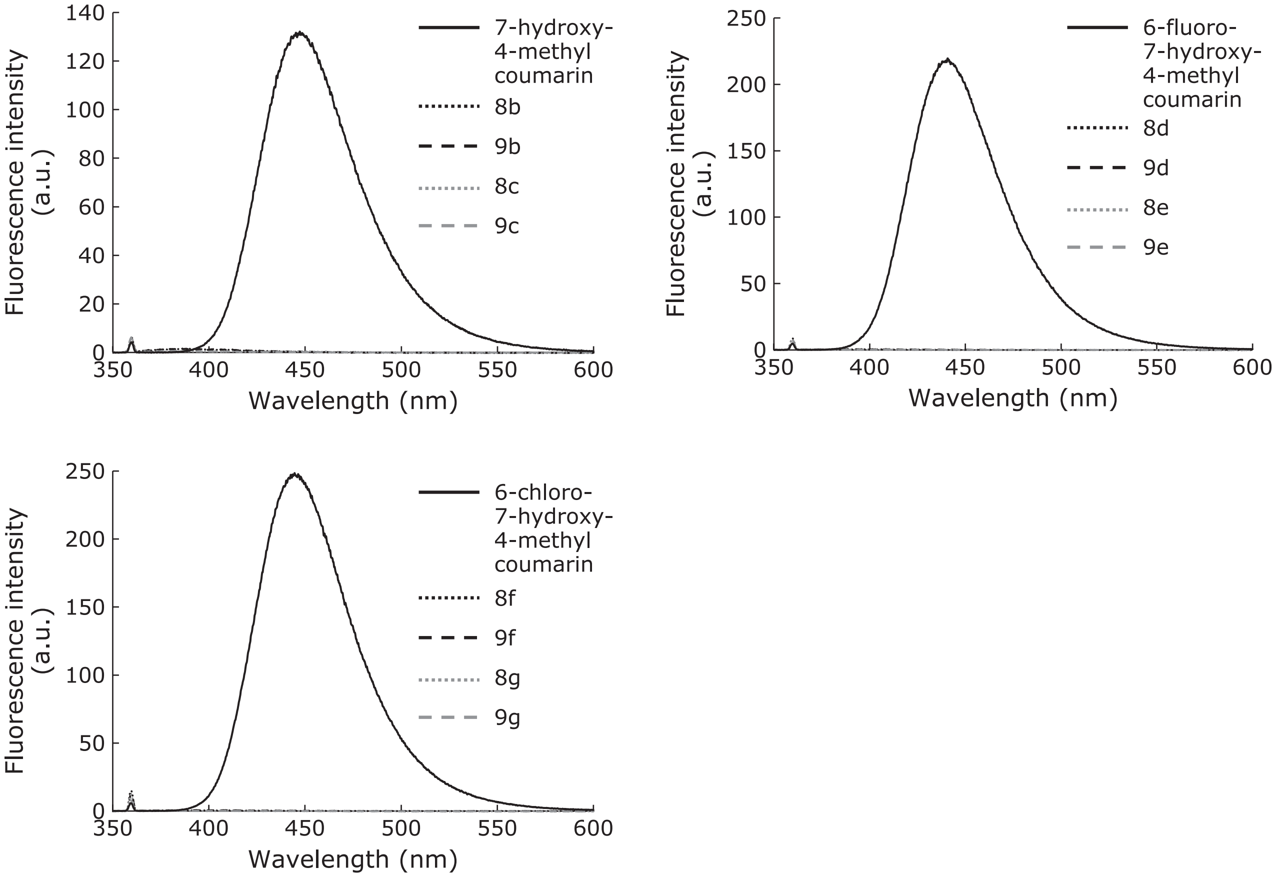

Supplemental Fig. 2. Fluorescence spectra of various 5 μM LSD1 substrates and reference compounds (coumarin derivatives) in 50 mM Tris-HCl buffer (pH 8.0). λ_{ex} = 360 nm.
