# Supplementary material for: Synthesis of artificial substrate based on inhibitor for detecting LSD1 activity
Source: J Clin Biochem Nutr. 2020 May 15;67(2):153–8. doi: 10.3164/jcbn.20-9 (PMC7533851; doi:10.3164/jcbn.20-9)
Supplement: Supplemental Figure 3 [file jcbn20-9sf03.pdf]

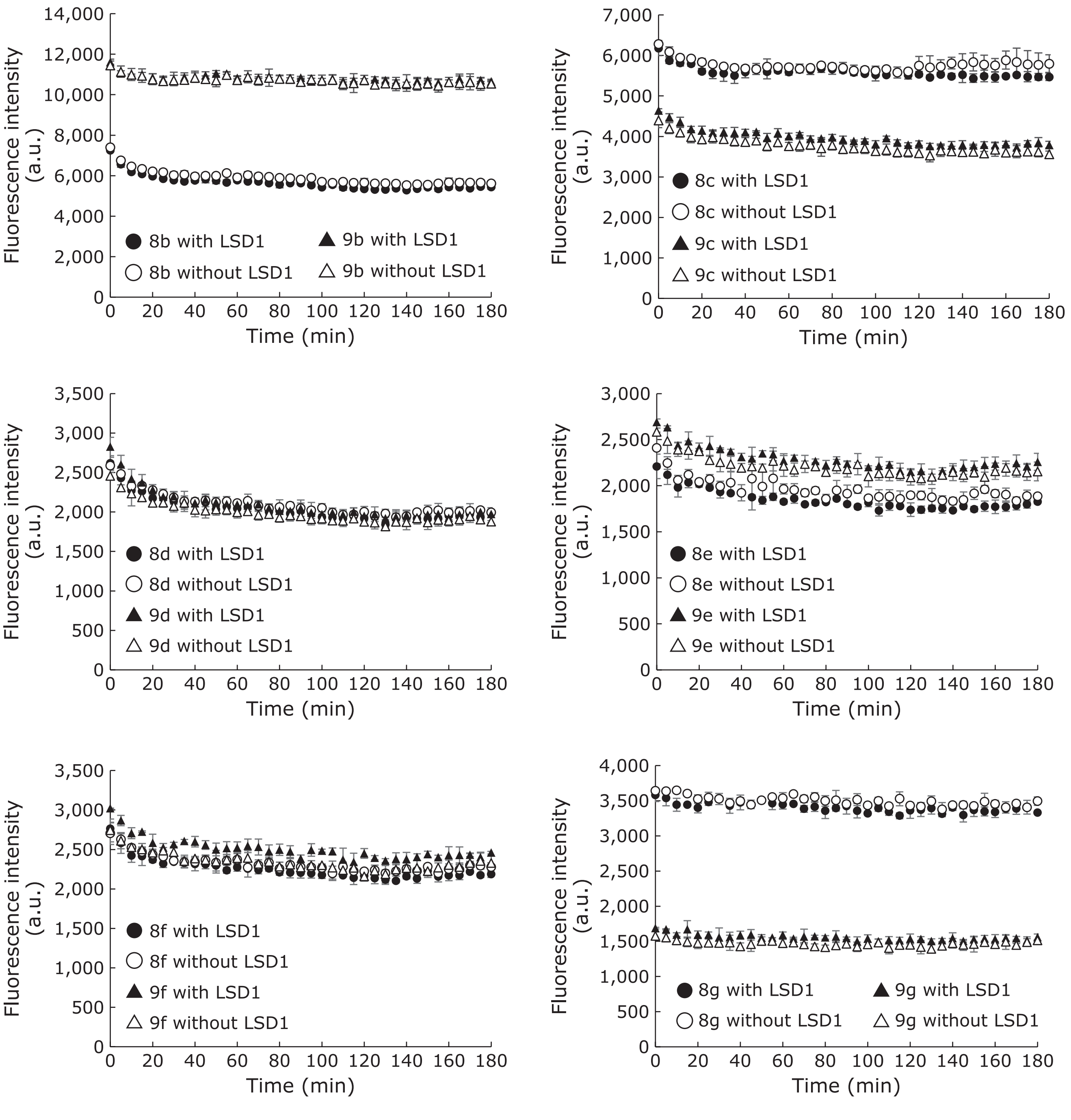

Supplemental Fig. 3. Enzymatic reactions of various LSD1 substrates with LSD1. Enzymatic reactions were performed in LSD1/HRP buffer, containing 25 μM LSD1 substrates in the presence or absence of 5 ng/μl LSD1. Fluorescence intensity was measured with ARVO X5 (filters; Ex. = 380/10 nm, Em. 460/20 nm) every 5 min for 3 h. The results are shown as mean ± SD (*n* = 3).
