# Supplementary material for: Synthesis of artificial substrate based on inhibitor for detecting LSD1 activity
Source: J Clin Biochem Nutr. 2020 May 15;67(2):153–8. doi: 10.3164/jcbn.20-9 (PMC7533851; doi:10.3164/jcbn.20-9)
Supplement: Supplemental Figure 4 [file jcbn20-9sf04.pdf]

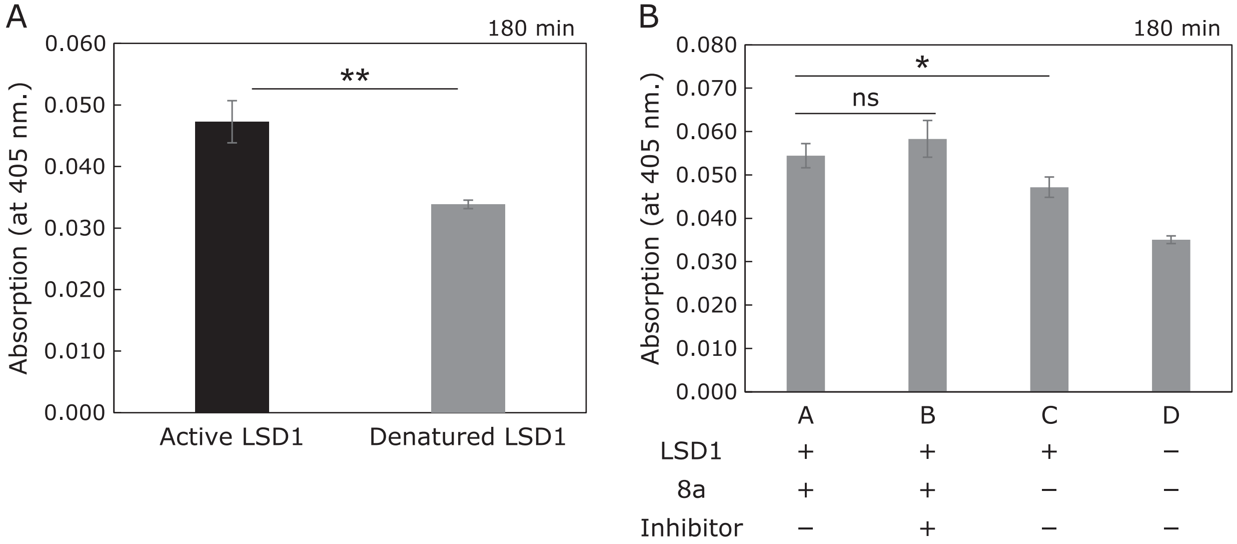

Supplemental Fig. 4. (A) Enzymatic reaction of 8a with active LSD1 or heat-denatured LSD1 (90°C for 5 min). Enzymatic reactions were performed in LSD1/HRP buffer, containing 25 μM 8a, 5 ng/μl LSD1 (active or denatured). Absorption was measured with ARVO X5 (filters; 405/10 nm) after 3 h incubation at 25°C. The results are shown as mean ± SD ($n = 3$). (B) Enzymatic reaction of 8a with LSD1 in the presence or absence of LSD1 inhibitor. Enzymatic reactions were performed in LSD1/HRP buffer, containing 25 μM 8a, 5 ng/μl LSD1 after pre-incubation in the presence or absence of 160 nM GSK-LSD1 for 30 min. Absorption was measured with ARVO X5 (filters; 405/10 nm) after 3 h incubation at 25°C. The results are shown as mean ± SD ($n = 3$). *$p<0.05$, **$p<0.01$, ns, not significant (Student's *t* test or Bonferroni-type multiple *t* test).
