# Supplementary material for: Synthesis of artificial substrate based on inhibitor for detecting LSD1 activity
Source: J Clin Biochem Nutr. 2020 May 15;67(2):153–8. doi: 10.3164/jcbn.20-9 (PMC7533851; doi:10.3164/jcbn.20-9)
Supplement: Supplemental Figure 5 [file jcbn20-9sf05.pdf]

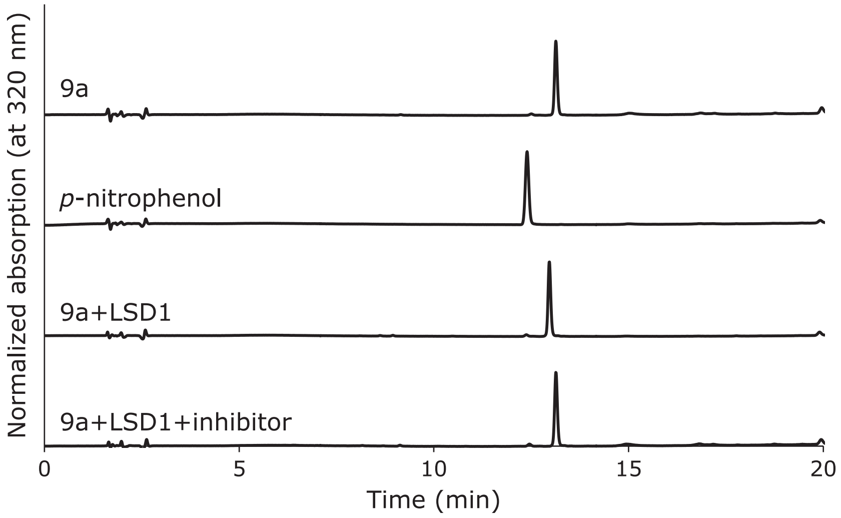

Supplemental Fig. 5. HPLC analysis of enzymatic reaction of LSD1 with 9a after 3 h. HPLC conditions: A:B = 90:10 (0 min) to 0:100 (20 min) with a linear gradient, A = 0.1% TFA and B = 0.1% TFA CH_3CN. Absorption at 320 nm was monitored.
